# Model-based closed-loop control of thalamic deep brain stimulation

**DOI:** 10.3389/fnetp.2024.1356653

**Published:** 2024-04-08

**Authors:** Yupeng Tian, Srikar Saradhi, Edward Bello, Matthew D. Johnson, Gabriele D’Eleuterio, Milos R. Popovic, Milad Lankarany

**Affiliations:** ^1^ Krembil Brain Institute—University Health Network, Toronto, ON, Canada; ^2^ Institute of Biomedical Engineering, University of Toronto, Toronto, ON, Canada; ^3^ KITE Research Institute, Toronto Rehabilitation Institute - University Health Network, Toronto, ON, Canada; ^4^ Department of Biomedical Engineering, University of Minnesota, Minneapolis, MN, United States; ^5^ Institute of Aerospace Studies, University of Toronto, Toronto, ON, Canada; ^6^ Center for Advancing Neurotechnological Innovation to Application, University Health Network and University of Toronto, Toronto, ON, Canada; ^7^ Department of Physiology, University of Toronto, Toronto, ON, Canada; ^8^ Institute of Medical Science, University of Toronto, Toronto, ON, Canada

**Keywords:** deep brain stimulation, closed-loop control (CLC) system, physiological model, short-term synaptic plasticity, thalamic ventral intermediate nucleus

## Abstract

**Introduction:** Closed-loop control of deep brain stimulation (DBS) is beneficial for effective and automatic treatment of various neurological disorders like Parkinson’s disease (PD) and essential tremor (ET). Manual (open-loop) DBS programming solely based on clinical observations relies on neurologists’ expertise and patients’ experience. Continuous stimulation in open-loop DBS may decrease battery life and cause side effects. On the contrary, a closed-loop DBS system uses a feedback biomarker/signal to track worsening (or improving) of patients’ symptoms and offers several advantages compared to the open-loop DBS system. Existing closed-loop DBS control systems do not incorporate physiological mechanisms underlying DBS or symptoms, e.g., how DBS modulates dynamics of synaptic plasticity.

**Methods:** In this work, we propose a computational framework for development of a model-based DBS controller where a neural model can describe the relationship between DBS and neural activity and a polynomial-based approximation can estimate the relationship between neural and behavioral activities. A controller is used in our model in a quasi-real-time manner to find DBS patterns that significantly reduce the worsening of symptoms. By using the proposed computational framework, these DBS patterns can be tested clinically by predicting the effect of DBS before delivering it to the patient. We applied this framework to the problem of finding optimal DBS frequencies for essential tremor given electromyography (EMG) recordings solely. Building on our recent network model of ventral intermediate nuclei (Vim), the main surgical target of the tremor, in response to DBS, we developed neural model simulation in which physiological mechanisms underlying Vim–DBS are linked to symptomatic changes in EMG signals. By using a proportional–integral–derivative (PID) controller, we showed that a closed-loop system can track EMG signals and adjust the stimulation frequency of Vim–DBS so that the power of EMG reaches a desired control target.

**Results and discussion:** We demonstrated that the model-based DBS frequency aligns well with that used in clinical studies. Our model-based closed-loop system is adaptable to different control targets and can potentially be used for different diseases and personalized systems.

## Introduction

Deep brain stimulation (DBS) is a standard therapy for various movement disorders, including Parkinson’s disease (PD) (Limousin et al., 1998), essential tremor (ET) (Dallapiazza et al., 2019), and dystonia (Hung et al., 2007). The thalamic ventral intermediate nucleus (Vim) is the primary surgical target of DBS for ET treatment. The stimulation frequency of clinical Vim–DBS for treating ET is usually chosen to be ≥ 130 Hz (Ondo et al., 1998; Dowsey-Limousin, 2002; Dembek et al., 2020). Currently, in clinics, DBS parameters—typically, frequency, amplitude, and pulse width—are usually manually tuned in a trial-and-error process, based on immediate clinical observations by neurologists (Deuschl et al., 2013; Grado et al., 2018; Boutet et al., 2021). Such manual DBS programming may be biased toward the neurologists’ expertise and patients’ experience, while requiring multiple clinical visits to test a large number of possible parameters, which cost time and induce stress in both patients and clinicians (Deuschl et al., 2013; Grado et al., 2018; Boutet et al., 2021). Additionally, manually programmed DBS delivers continuous DBS (cDBS) to the patient, which can cause side effects and exacerbate stimulation habituation (Pahwa et al., 2006; Barbe et al., 2011; Cernera et al., 2021). Continuous stimulation can also decrease battery life, thus increasing patients’ burden caused by battery replacement surgeries or battery recharging processes (Opri et al., 2020; Khaleeq et al., 2019). Hence, there is a need for a control system that can automatically adjust DBS parameters in a closed-loop fashion. Such closed-loop DBS needs to be based on a biomarker that characterizes the patient’s clinical states.

A closed-loop DBS control system consists of three essential components: (*i*) input DBS pulses; (*ii*) output feedback, i.e., the biomarker observed during DBS; and (*iii*) feedback control, which adjusts the DBS parameters based on the feedback biomarker (Arlotti et al., 2016; Little and Brown, 2012; Grado et al., 2018). Such a system offers an automatic way to adapt stimulation parameters moment-to-moment with respect to the patient’s clinical states (Arlotti et al., 2016). Compared with manually programmed (open-loop) cDBS, closed-loop DBS can significantly reduce the stimulation time and enhances clinical efficacy (Arlotti et al., 2016; Little et al., 2013; Cernera et al., 2021).

Most common closed-loop DBS systems use local field potential (LFP), recorded from stimulated nuclei, to find an effective feedback biomarker (Little et al., 2013; Priori et al., 2013; Velisar et al., 2019), e.g., the power of the beta oscillation (12–32 Hz) of LFP recorded in the subthalamic nucleus (STN) for reducing PD symptoms (Grado et al., 2018; Arlotti et al., 2016; Little et al., 2013). Velisar et al. (2019) developed a closed-loop DBS control system in which the beta oscillation power of the STN-LFP was chosen as the biomarker and the DBS amplitude was updated by a dual-threshold control method that maintains the STN–LFP beta power within a certain range. Other signals like muscle activities in electromyography (EMG) or inertial measurement units (IMUs) have also been used as biomarkers in treatment of tremors by closed-loop DBS (Cernera et al., 2021; Yamamoto et al., 2013; Haddock et al., 2017; Herron et al., 2017). For example, in the treatment of ET, Herron et al. (2017) developed a closed-loop DBS system that controls the EMG power to be below a specified threshold. There are also other types of feedback biomarkers used in closed-loop DBS, e.g., single-unit recordings (Rosin et al., 2011) and the coherence among electroencephalogram (EEG) recordings (Silberstein et al., 2005).

Regardless of the type of the feedback biomarker, DBS settings are determined solely based on neural (e.g., LFP) or behavioral (e.g., EMG) signals in most existing closed-loop DBS controllers (Velisar et al., 2019; Little et al., 2013; Herron et al., 2017; Haddock et al., 2019; Castaño-Candamil et al., 2020; Chandrabhatla et al., 2023). However, these methods suffer from the lack of an understanding of the physiological mechanisms underlying the DBS and disease-related neuronal circuits. An effective approach to overcome this problem is to embed a computational model of the underlying mechanisms into the control system (Grado et al., 2018). For example, to control Parkinson’s disease, closed-loop DBS systems were developed based on the physiological models of the related cortico-basal ganglia–thalamic network (Liu et al., 2021; Fleming et al., 2020). Liu et al. (2021) used the control system to suppress the beta oscillations in the cortex, and Fleming et al. (2020) suppressed the beta power of LFP in the STN. Although these computational studies included methods for adjusting DBS in a closed-loop manner (Fleming et al., 2020; Grado et al., 2018; Liu et al., 2021), the models used were not validated for replicating/tracking experimental data nor did they incorporate DBS mechanisms of actions, e.g., DBS-induced short-term synaptic plasticity (Milosevic et al., 2021; Tian et al., 2023a; Ghadimi et al., 2022).

In this work, we develop a closed-loop control system to adjust the stimulation frequency of Vim–DBS automatically. Our control system is based on a computational model that predicts the EMG activities in response to different frequencies of Vim–DBS. In this computational model, the firing rate of Vim neurons in response to Vim–DBS is predicted by our recently developed rate network model that reproduces the human clinical data recorded in Vim neurons in response to different DBS frequencies (10–200 Hz) (Tian et al., 2023b). Dynamics of DBS-induced short-term synaptic plasticity (Tian et al., 2023b) are incorporated in the rate network model. We used a neural model simulation study including models of DBS, Vim, the motor cortex, motoneurons in the spinal cord, and muscle fibers to generate muscle activities (represented by EMG). To link Vim–DBS to EMG signals in our model-based control framework, model-predicted EMG signals, generated in our simulation study, are used to calculate the feedback biomarker by a polynomial fit, which is processed and implemented in a proportional–integral–derivative (PID) controller (O’Hara et al., 1997; Raj et al., 2016; Sattar et al., 2019) that automatically updates the appropriate DBS frequency. Our model-predicted EMG can predict the symptoms of essential tremor during DBS-OFF and is consistent with clinical observations of tremors during different frequencies of Vim–DBS. In a closed-loop DBS control system, the ability of predicting the biomarker decreases the probability of delivering inappropriate DBS frequencies to the patient, and thus increases the therapeutic efficacy and reduces side effects.

The proposed model-based closed-loop DBS control system is based on synthetic EMG data and is currently in the stage of proof-of-concept. However, we anticipate that our computational framework can facilitate the development of model-based control systems that can be potentially implemented in and out of the clinic to automatically update the appropriate DBS frequency for individual patients suffering from different diseases.

## Materials and methods

We developed a computational framework for incorporating the physiological mechanisms of deep brain stimulation into controlling disease symptoms. This framework consists of two main parts: (1) a computational model characterizing the physiological mechanism of the stimulated neuronal network and (structurally/functionally) connected neurons and (2) a feedback control reflecting the disease state. In this study, we use computer simulations and apply our framework to control DBS frequency for reducing ET symptoms observed from EMG signals.

### Computational model

The computational model consists of four components: (*i*) neural activities, spikes, generated by the Vim network model in response to different DBS frequencies; (*ii*) motor cortex neural activities influenced by propagation of Vim–DBS effects to the motor cortex; (*iii*) spinal motoneuron activities impacted by neurons in the motor cortex; (*iv*) motor unit action potentials in the muscle fibers innervated by the spinal motoneurons.

#### (i) Vim network model impacted by Vim–DBS

The baseline firing rate of Vim neurons during DBS-OFF is often chosen in the range of 10–40 Hz (Milosevic et al., 2021; Tian et al., 2023b). The firing rate dynamics of the Vim neurons in response to Vim–DBS were simulated by our previous model of the Vim network based on clinical DBS data recorded during surgery on human patients with essential tremor (ET) (Supplementary Method S2) (Tian et al., 2023b). The impact of DBS pulses was modeled as the induction of synaptic release (Tian et al., 2023b), the dynamics of which is characterized by the Tsodyks and Markram (TM) model (Tsodyks et al., 1998) (Supplementary Method S1) of short-term synaptic plasticity (STP) (Milosevic et al., 2021). DBS pulses are fed into the TM model to obtain the post-synaptic currents into Vim neurons (Tian et al., 2023b). The firing rate network model consists of recurrent connections among three neural groups: DBS-targeted Vim neurons, external excitatory nuclei, and inhibitory nuclei (Supplementary Method S2) (Tian et al., 2023b). Our previous model could accurately reproduce the clinically recorded instantaneous firing rate of the Vim neurons receiving DBS of different stimulation frequencies (10–200 Hz) (Supplementary Figure S5) (Tian et al., 2023b).

#### (ii) Propagation to the primary motor cortex

In our model, the effects of Vim–DBS are propagated to the primary motor cortex (M1). We modeled the propagation using the dynamics induced by two sources: (1) the effects of Vim–DBS and (2) the background neuronal activities that induce tremor symptoms. The Vim–DBS effects are induced by the direct DBS activation of the axons projected to the M1 neuron and the firings of the Vim neurons during Vim–DBS.

These effects consist of direct axon activation and DBS-induced Vim firings. DBS activates the axons connecting to the synapses projecting to the M1 neuron, and these synapses are characterized by the Tsodyks and Markram model (Tsodyks et al., 1998) (Supplementary Method S1). In addition to the direct axon activation, the M1 neuron is also affected by the DBS-induced firings of the Vim neurons. With our previous Vim network model (Tian et al., 2023b), we simulated the instantaneous firing rate of the Vim neurons receiving DBS of different stimulation frequencies (10–200 Hz). The Vim firing rate signal is the time-varying Poisson rate for generating Poisson spike trains, which were passed to the TM-modeled synapses to produce the post-synaptic current in the M1 neurons (Supplementary Method S1).

In addition to the DBS effects, we also modeled the background neuronal activities inducing tremor symptoms. The tremor activities observed in the EMG from ET patients are often in the frequency band of 4–8 Hz (Halliday et al., 2000; Hess and Pullman, 2012; Herron et al., 2017). The tremor-inducing background firing rate was taken as a waveform consisting of 6-Hz bursts with a baseline shift (Supplementary Figure S1). To be consistent with the EMG recordings from ET patients (Halliday et al., 2000; Vaillancourt et al., 2003; Hess and Pullman, 2012; Zhang et al., 2017), each burst consists of three consecutive sinusoidal waves and the period of each wave is 20 ms (Supplementary Figure S1). We then generated Poisson spike trains from the background firing rate waveform; these spikes were then passed to the M1 synapses characterized by the TM model (Tsodyks et al., 1998), which produced the post-synaptic current into the M1 neurons (Supplementary Method S1).

The membrane potential of one neuron in the M1 neuron population was characterized by a leaky integrate-and-fire (LIF) model (Eq. 1) as follows:

$$\begin{array}{c} \frac{dV}{dt}=\frac{-\left(V-E_{L}\right)+RI_{syn}}{\tau_{V}}\\ I_{syn}=I_{DBS}+I_{b} \end{array},$$ (1)

where $E_{L}=-$ 65 mV is the equilibrium potential, R (resistance parameter) = 1 MΩ, and $\tau_{V}$ = 10 ms is the membrane time constant; spikes occur when $V\geq V_{th}$ , where $V_{th}$ = − 35 mV. The reset voltage is − 90 mV, and the absolute refractory period is 1 ms. $I_{syn}$ is the total post-synaptic input current, consisting of the inputs induced by Vim-DBS ( $I_{DBS}$ ) and the background inputs generating the tremor ( $I_{b}$ ), and was obtained by the TM model (Tsodyks et al., 1998) that incorporates all the input spikes (see Supplementary Method S1).

#### (iii) Projection from the primary motor cortex to spinal motoneurons

We modeled the effects of Vim–DBS as being propagated to a population of 150 M1 neurons ( $N_{c}$ = 150), which project to 120 motoneurons ( $N_{m}$ = 120) in the spinal cord (Moezzi et al., 2018; Watanabe et al., 2013). We assumed that each motoneuron randomly connects to 70 M1 neurons and receives monosynaptic inputs from each M1 neuron (Moezzi et al., 2018; Porter et al., 1995). Following a spike from an M1 neuron, we modeled the post-synaptic current into a motoneuron by the rule of spike-timing-dependent plasticity (STDP) from Izhikevich (2006):

$$i\left(t\right)=C\;e^{-\frac{t-t_{d,cm}-k}{\;\tau_{i}}},$$ (2)

where k is a spike timing of an M1 neuron, $t_{d,cm}$ = 10 ms is the M1-to-spinal-motor-neuron transmission delay (Baker and Lemon, 1998), $t\geq k+t_{d,cm}$ is a time point following the M1 spike timing k , $\tau_{i}$ = 20 ms is a time constant, and C = 0.1 nA is the scaling factor (Izhikevich, 2006). The membrane potential of the motoneuron is given by an LIF model (Eq. 3) equivalent to that of Herrmann and Gerstner (2002) (Herrmann and Gerstner, 2002; Moezzi et al., 2018):

$$V_{j}\left(t\right)=V_{0}e^{-\frac{t-t_{j}^{sp}}{\tau_{p}}}h\left(t-t_{j}^{sp}\right)+\frac{R_{m}}{\tau_{m}}\left(1-e^{-\frac{t-t_{j}^{sp}}{\tau_{r}}}\right)\int_{0}^{t-t_{j}^{sp}}e^{-\;\frac{s}{\tau_{m}}}\;I_{j}\left(t-s\right)ds,$$ (3)

where $t_{j}^{sp}$ is the last spike timing of the *j* ^th^ motoneuron. h(t) is the Heaviside step function, which is 0 when its argument is negative and 1 otherwise. $I_{j}\left(t\right)$ is the post-synaptic current from M1 neurons into the *j* ^th^ motoneuron and is a summation of $i\left(t\right)$ (Eq. 2) from each of the 70 M1 neurons projecting to the motoneuron. $V_{0}=-22$ mV is the reset membrane potential (Moezzi et al., 2018). When the membrane potential reaches a firing threshold $V_{th}$ , it is instantaneously reset to $V_{0}$ , and the integration process restarts. For each motoneuron, the firing threshold $V_{th}$ ∈ [5, 15] mV is chosen randomly (Moezzi et al., 2018). $R_{m}$ = 36 M Ω is the input resistance (Moezzi et al., 2018). $\tau_{p}$ = 2 ms is the refractory time constant. $\tau_{m}$ = 4 ms is the passive membrane time constant. $\tau_{r}$ = 100 ms is the recovery time constant (Borg and Borg, 1987; Moezzi et al., 2018). The firing rate of a human motoneuron is normally between 5 and 50 Hz (Macefield et al., 1993), although it can be over 100 Hz for a brief period during fast contractions (Duchateau and Baudry, 2014).

#### (iv) Generation of EMG activities from spinal motoneuron spikes

The spikes from the spinal motoneurons generate action potentials in the motor units of muscle fibers (Moezzi et al., 2018; Watanabe et al., 2013). These motor unit action potentials (MUAPs) were modeled (Eq. 4) as follows (Watanabe et al., 2013; Moezzi et al., 2018; Lo Conte et al., 1994):

$$M_{j}\left(t\right)=A_{j}\left(t-\tau_{j}^{sp}\right)e^{-{\left(\frac{t-\tau_{j}^{sp}}{\lambda }\right)}^{2}}h\left(t-\tau_{j}^{sp}\right);\;\tau_{j}^{sp}=t_{j}^{sp}+t_{d,mm},$$ (4)

where $M_{j}$ is the MUAP of the *j* ^th^ motor unit, corresponding to the *j* ^th^ motoneuron; λ= 2 ms is the time factor (Moezzi et al., 2018); $t_{j}^{sp}$ is the spike timing; $t_{d,mm}$ = 10 ms is the motor-neuron-to-muscle conduction delay in humans (Eyre et al., 2000); and *h(t)* is the Heaviside step function. $A_{j}$ is the scale factor of the amplitude of activities in the *j* ^th^ motor unit (Li et al., 2012; Moezzi et al., 2018) and was modeled as following the exponential distribution $A_{j}\;∼\;\mathrm{Exp}\;\left(\left(\frac{1}{\mu }\right)\right.$ , where μ is the mean of distribution (Li et al., 2012; Moezzi et al., 2018); we chose μ= 7 × 10^–3^ to be consistent with the EMG simulation during transcranial magnetic stimulation (TMS), as given in Moezzi et al. (2018). Finally, the surface EMG ( $y\left(t\right)$ ) was modeled (Eq. 5) as the summation of MUAPs with a low-level Gaussian white noise ( $\varepsilon \left(t\right)$ ) (Watanabe et al., 2013; Moezzi et al., 2018) with a standard deviation of 0.025 mV:

$$y\left(t\right)=\varepsilon \left(t\right)+\sum_{j}M_{j}\left(t\right).$$ (5)

### Feedback control for DBS frequency

Our computational model simulates the EMG signal in response to different DBS frequencies. Simulated EMG signals are used to calculate the feedback biomarker which controls the DBS frequency. The feedback control consists of three main parts: (1) biomarker identification, (2) computation of a system output from the biomarker, and (3) a closed-loop controller that implements the system output to update the DBS frequency.

### Biomarker identification

The EMG simulation of our computational model is slow to implement: it takes more than 30 min when using MATLAB R2022b with a personal computer to simulate 10 s of the EMG signal. Thus, to facilitate the implementation speed of the model in a closed-loop control system, we need rapid EMG estimation to replace the direct EMG simulation. We implemented a polynomial method to estimate the EMG from the Vim firing rate, and the direct model-simulated EMG is used as the reference (i.e., reference EMG) for estimation. We implemented the MATLAB custom function *polyfit* for polynomial estimation, which gives a least-square fit of the polynomial coefficients. The estimated EMG is formulated as follows:

$$\begin{array}{c} \hat{y}\left(t\right)=\psi \left(\zeta \left(x\left(t\right)\right)\right)+\varphi_{0}+\varepsilon \left(t\right)\\ \psi \left(\zeta \left(x\left(t\right)\right)\right)=\sum_{n=1}^{N}\varphi_{n}{\left[\zeta \left(x\left(t\right)\right)\right]}^{n}\text{ and }\zeta \left(x\left(t\right)\right)=\frac{x\left(t\right)\;-\;\bar{x}\left(t\right)}{sd\left[x\left(t\right)\right]} \end{array},$$ (6)

where $x\left(t\right)$ is the Vim firing rate simulated from our previous Vim network model (Tian et al., 2023b) and $\hat{y}\left(t\right)$ is the estimated EMG. $\zeta \left(x\left(t\right)\right)$ is the standardization of $x\left(t\right)$ ; $\bar{x}\left(t\right)$ and $sd\left[x\left(t\right)\right]$ are the mean and standard deviation of $x\left(t\right)$ , respectively. Then, $\zeta \left(x\left(t\right)\right)$ has a mean of 0 and a standard deviation of 1. The polynomial order is 25, and $\left.{\left\{\varphi \right.}_{0},\varphi_{1},\ldots ,\varphi_{N},N=25\right\}$ are the polynomial coefficients. The $R^{2}$ statistic (Colin Cameron and Windmeijer, 1997) of the fit generally increases with increase in the polynomial order (Supplementary Figure S3), and 25 is a minimal order of the polynomial when $R^{2}$ converges. $\varepsilon \left(t\right)$ is the Gaussian white noise with a standard deviation of 0.038 mV. We fitted the consistent polynomial coefficients (Supplementary Table S2) across data from different DBS frequencies, including 10, 50, 80, 100, 120, 130, 140, 160, and 200 Hz. Our previous work showed that consistent model parameters fitted based on concatenated DBS frequencies in a certain range—in this case, [10–200] Hz—can be consistently applied to unobserved frequencies (e.g., 25 and 180 Hz) in the same range (Tian et al., 2023a). Thus, we implement the polynomial-estimated EMG as the biomarker to control the DBS frequencies in the range [10–200] Hz.

### Computation of system output from the estimated EMG

The power spectral density (PSD) of the estimated EMG $\hat{y}\left(t\right)$ (Eq. 6) is the system output for updating the frequency of the input DBS (10–200 Hz). In the system output, we consider PSD in the frequency band [2–200] Hz, which includes the frequencies of both DBS and EMG activities (Halliday et al., 2000; Hess and Pullman, 2012; Herron et al., 2017; Milosevic et al., 2021). The power density is calculated by the following equation (Miller, 2019; Liu et al., 2021), with a sampling frequency of 0.5 kHz:

$$p\left(f,t,u\right)={\left|\frac{1}{\sqrt{w}}\int_{-\frac{w}{2}}^{\frac{w}{2}}{\hat{y}}_{u}\left(t+s\right)H\left(s\right)e^{i2\pi fs}ds\right|}^{2};\text{ furthermore},$$ (7)

$H\left(s\right)=\frac{1}{2}\left(1+\cos\left(\frac{2\pi s}{w}\right)\right)$ .

where f represents the frequency of the EMG activities. $H\left(s\right)$ is the Hann windowing function (Miller, 2019; Porat, 1997), and w is the width of the window, chosen to be w = 1 s to ensure stability (Liu et al., 2021; Miller, 2019). ${\hat{y}}_{u}\left(t\right)$ represents the estimated EMG in response to DBS with stimulation frequency u . The total power of EMG activities over the initial period T of the estimated EMG signal is then given as

$$P\left(f,T,u\right)=\int_{0}^{T}p\left(f,t,u\right)dt.$$ (8)

The EMG power thus computed can be used to analyze the EMG activities in the frequency domain in response to different frequencies of DBS.

In response to DBS frequency u , the PSD of EMG within the frequency band of [2–200] Hz is approximated as the sum of the power density ( $p\left(f,t,u\right)$ , Eq. 7) from each integer DBS frequency (2, 3, 4, …, 200 Hz) in [2–200] Hz, and the system output $z\left(u\right)$ is calculated as the approximated PSD in the initial T = 5 s of the estimated EMG:

$$z\left(u\right)=\frac{1}{T}\int_{0}^{T}\sum_{f=2}^{200}p\left(f,t,u\right)dt.$$ (9)

Finally, a closed-loop controller updates the DBS frequency u so that $z\left(u\right)$ is close to a specified control target $\beta_{z}$ (see the next section).

#### The PID controller that updates the DBS frequency

We implemented the proportional–integral–derivative (PID) controller (O’Hara et al., 1997; Raj et al., 2016; Sattar et al., 2019) to update the DBS frequency based on the system output, *z* (Eq. 9). The updated DBS frequency $u\left(t\right)$ is computed as Eq. 10

$$\left\{\begin{array}{c} u\left(t_{m+1}\right)=K_{p}e\left(t_{m}\right)+K_{i}\sum_{j=1}^{m}e\left(t_{j}\right)∆t+K_{d}\frac{e\left(t_{m}\right)-e\left(t_{m-1}\right)}{∆t}\\ e\left(t_{m}\right)=z\left(u\left(t_{m}\right)\right)-\beta_{z} \end{array}\right.$$ (10)

and is evaluated at the time points { $t_{1}$ , $t_{2}$ , …, $t_{M}$ }, with $t_{1}$ = 0, $t_{M}$ = 10 min, and ∆t = $t_{m}$ – $t_{m-1}$ = 1/3 min. The simulation of the PID controller was performed with MATLAB R2022b. $K_{p}$ , $K_{i}$ , and $K_{d}$ denote the proportional, integral, and derivative gains, respectively. The error signal $e\left(t\right)$ is the difference between the system output *z* and its control target $\beta_{z}$ .

## Results

**FIGURE 1:**
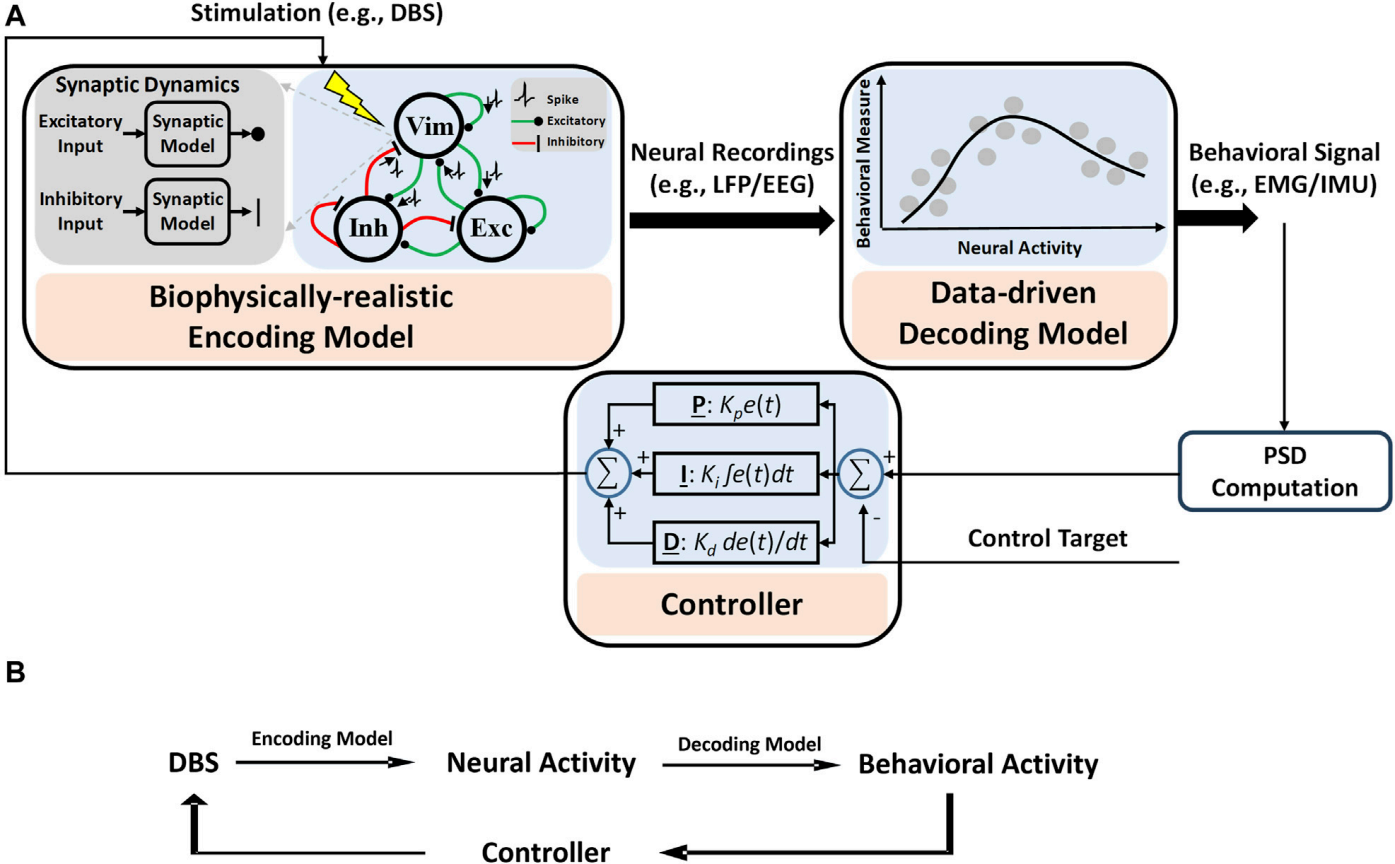
Computational framework for the proposed model-based closed-loop DBS control system. **(A)** Biophysical details, e.g., dynamics of synaptic plasticity, are preserved in the encoding model to identify how different patterns of DBS change the neural activities of simulated neurons. A data-driven decoding model was used to map the neural activity to behavioral signals like EMG. A controller is used to adjust DBS in a closed-loop manner. The control target is a specified value of the system output, which is related to the power spectral density (PSD) of EMG (see Eq. 10 for details). **(B)** A summary diagram of proposed computational framework.

**FIGURE 2:**
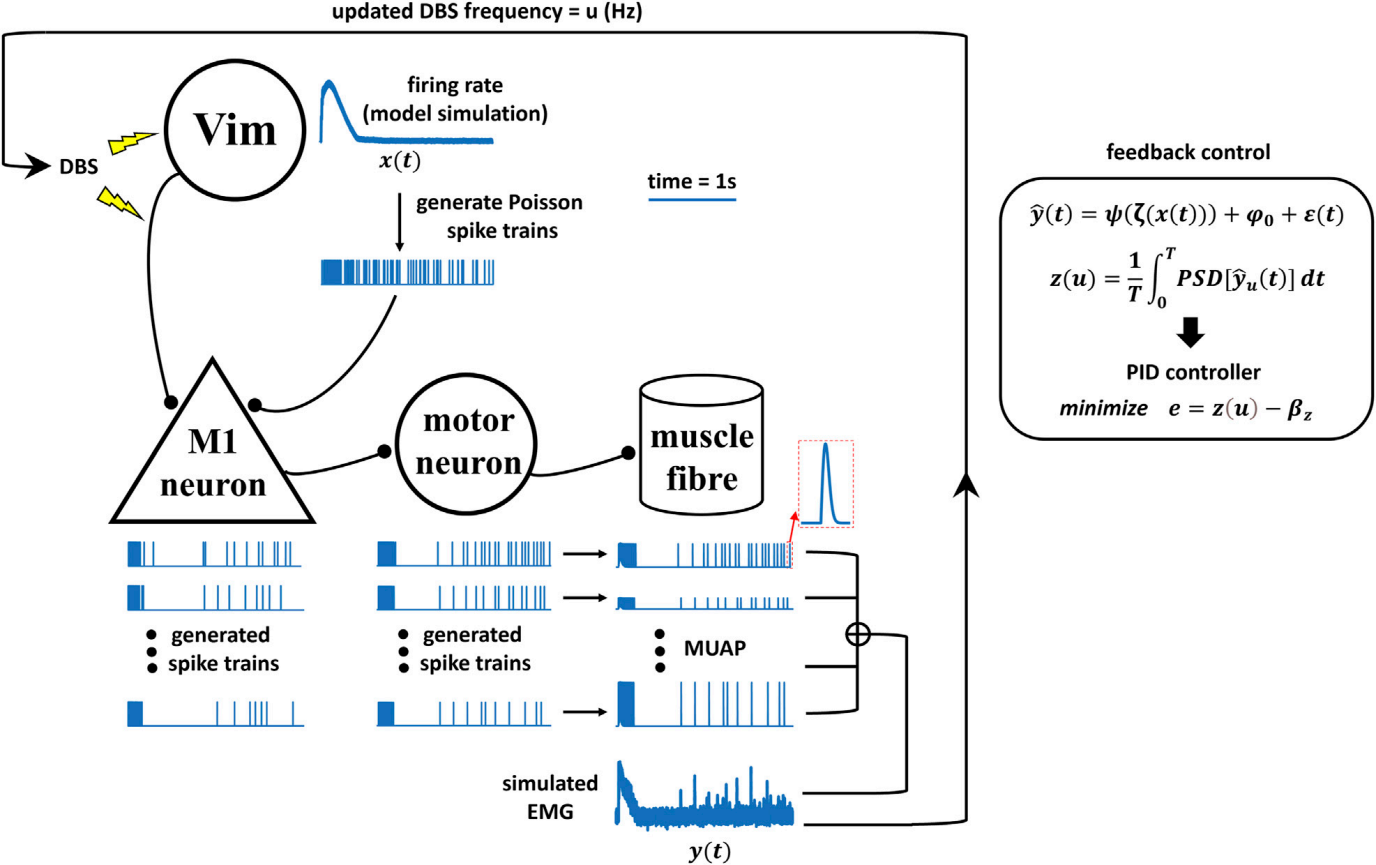
Schematics of the neural model simulation study for Vim–DBS control.

Our proposed computational framework for model-based closed-loop DBS control is shown in Figure 1. As shown in Figure 1A, an encoding model is used to replicate (predict) neuronal activities in response to DBS frequencies. In this encoding model, the dynamics of synaptic plasticity and other biophysical details can be preserved, and model parameters are estimated by fitting the model output to experimental data (Tian et al., 2023b; Tian et al., 2023a). Additionally, to map neural activities to behavioral signals, we use a data-driven approach (Eq. 6) in our framework. A controller, e.g., a PID controller, is developed to adjust DBS patterns given behavioral signals solely. It is to be noted that, unlike the encoding model, using neural models to identify neural–behavioral relationships requires building a network of several neuronal circuits (see our simulation study in Figure 2), which in turn increases the complexity of this type of model for mapping neural features to behavioral activities. However, we show that data-driven approaches are strong alternatives. The proposed computational framework in Figure 1B highlights the contributions of encoding (biophysical) and decoding (data-driven) models.

In the next sections, we present details of the model-based closed-loop DBS system that can effectively control the DBS frequency based on EMG signals generated by a neural model simulation (Figure 2) of the underlying neuronal network (from DBS/Vim neural activity to muscle activity).

### Schematics of the neural model simulation study for Vim-DBS control

The simulation study for the Vim–DBS control system is schematized in Figure 2. The effects of Vim–DBS included direct axon activation and DBS-induced Vim firings, which were simulated from our previous Vim network model established based on clinical Vim–DBS data (Tian et al., 2023b). The Vim–DBS effects are propagated to the M1 neuron, which projects to the motoneuron in the spinal cord. The firings of the spinal motoneurons innervate the corresponding motor units in the muscle fibers and induce motor unit action potentials (MUAPs). The simulated EMG consists of a linear summation of MUAPs and a low-level Gaussian white noise. In the feedback control, in order to facilitate the implementation speed, we estimated the simulated EMG by a polynomial fit. Then, we computed the mean power spectral density (PSD) of such polynomial-estimated EMG as the system output. Finally, a proportional–integral–derivative (PID) controller updated the DBS frequency that brought the system output close to a specified control target.

DBS pulses are delivered to the neurons in the thalamic ventral intermediate nucleus (Vim) and also activate the axons projecting to neurons in the primary motor cortex (M1). The firing rate of the Vim neurons impacted by DBS was obtained from our previous Vim network model that reproduced experimental DBS data (Tian et al., 2023b). Spike trains are generated from the modeled Vim firing rate (* $x\left(t\right)$ *) as a Poisson process and are propagated to the M1 neurons. The spikes from the M1 neurons are then propagated to the motoneurons in the spinal cord. The spikes from these motoneurons innervate the corresponding motor units in the muscle fibers and induce motor unit action potentials (MUAPs). The simulated EMG (* $y\left(t\right)$ *) is a linear summation of MUAPs with additive low-level Gaussian white noise. In the feedback control, we estimate the simulated EMG by fitting a polynomial function *ψ* (Eq. 6), and this estimated EMG (* $\hat{y}\left(t\right)$ *) is the biomarker used in the control. *$\varphi_{0}$* is the constant term of *ψ , $\varepsilon \left(t\right)$* is the Gaussian white noise, and *$\zeta \left(x\left(t\right)\right)$* is the standardization of the modeled Vim firing rate *$x\left(t\right)$* (Eq. 6). The system output *$z\left(u\right)$* is calculated as the mean power spectral density (PSD) over the initial *T* of the biomarker *$\hat{y}\left(t\right)$* (Eq. 9). Finally, a proportional–integral–derivative (PID) controller (Eq. 10) updates the DBS frequency u that reduces the error (* e *) between the system output *$z\left(u\right)$* and a specified control target *$\beta_{z}$ *.

### The Vim–Cortex propagation

**FIGURE 3:**
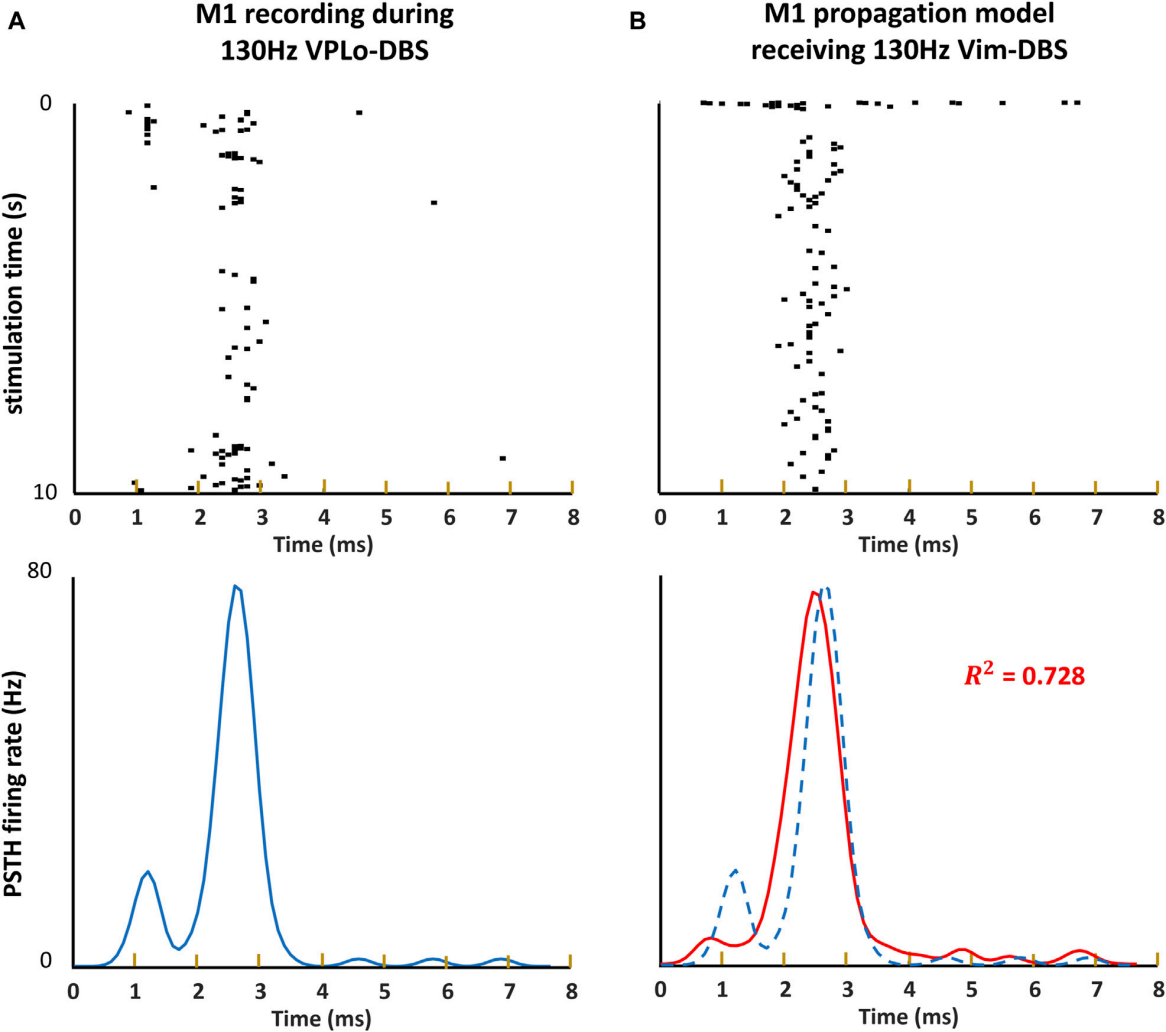
Raster plot and PSTH of model simulation and non-human primate recording. **(A, B)** The spike times occurring within each inter-pulse interval during 10 s of 130-Hz DBS are visualized as a raster plot. We obtain an estimate of the instantaneous firing rate induced around each DBS event by computing a peristimulus time histograms (PSTHs), convolving the spikes with a 0.2-ms Gaussian kernel. **(A)** T*he raster plot and PSTH of the non-human primate single-unit recording in the primary motor cortex (M1) during 130-Hz VPLo-DBS* (Agnesi et al., 2015). **(B)** The raster plot and PSTH of our model simulation of M1 spikes during 130-Hz Vim-DBS. We compute the $R^{2}$ statistic that compares the model simulation (solid line) with experimental data (dashed line).

Our model of the propagation of the Vim–DBS effects to the cortical M1 neurons was validated by using a 10-s block of neural spike data from single-unit recordings in M1 during 130-Hz VPLo-DBS in non-human primates, reported in Agnesi et al. (2015). The thalamic VPLo nucleus (ventralis posterior lateralis pars oralis, in the Olszewski atlas) in non-human primates is homologous to the Vim in humans (Molnar et al., 2005; Xiao et al., 2018). To assess the response of Vim to 130-Hz DBS, a peristimulus time histogram (PSTH) was calculated based on spike times occurring between 0 and 7.7 ms after each DBS pulse, with the PSTH further smoothed by an optimal Gaussian kernel (Shimazaki and Shinomoto, 2007; Shimazaki and Shinomoto, 2010) (Figure 3), both for empirical and simulated data. The standard deviation of the Gaussian kernel was 0.2 ms, which was obtained from optimization of the Gaussian kernel to best characterize the spikes using a Poisson process (Shimazaki and Shinomoto, 2007; Shimazaki and Shinomoto, 2010; Tian et al., 2023b). Our Vim–M1 propagation model’s generated M1 spike activity behaved similarly to that of the empirically recorded non-human primate M1, as indicated by the spike raster plot and PSTH firing rate analysis (Figure 3). We computed the $R^{2}$ statistic (Colin Cameron and Windmeijer, 1997) to compare model-simulated and experimental PSTHs, and $R^{2}$ = 0.728 represents a good model fit (Figure 3B). Thus, our Vim–M1 propagation model could reflect the M1 dynamics during 130-Hz Vim–DBS.

### EMG simulation from the computational model

**FIGURE 4:**
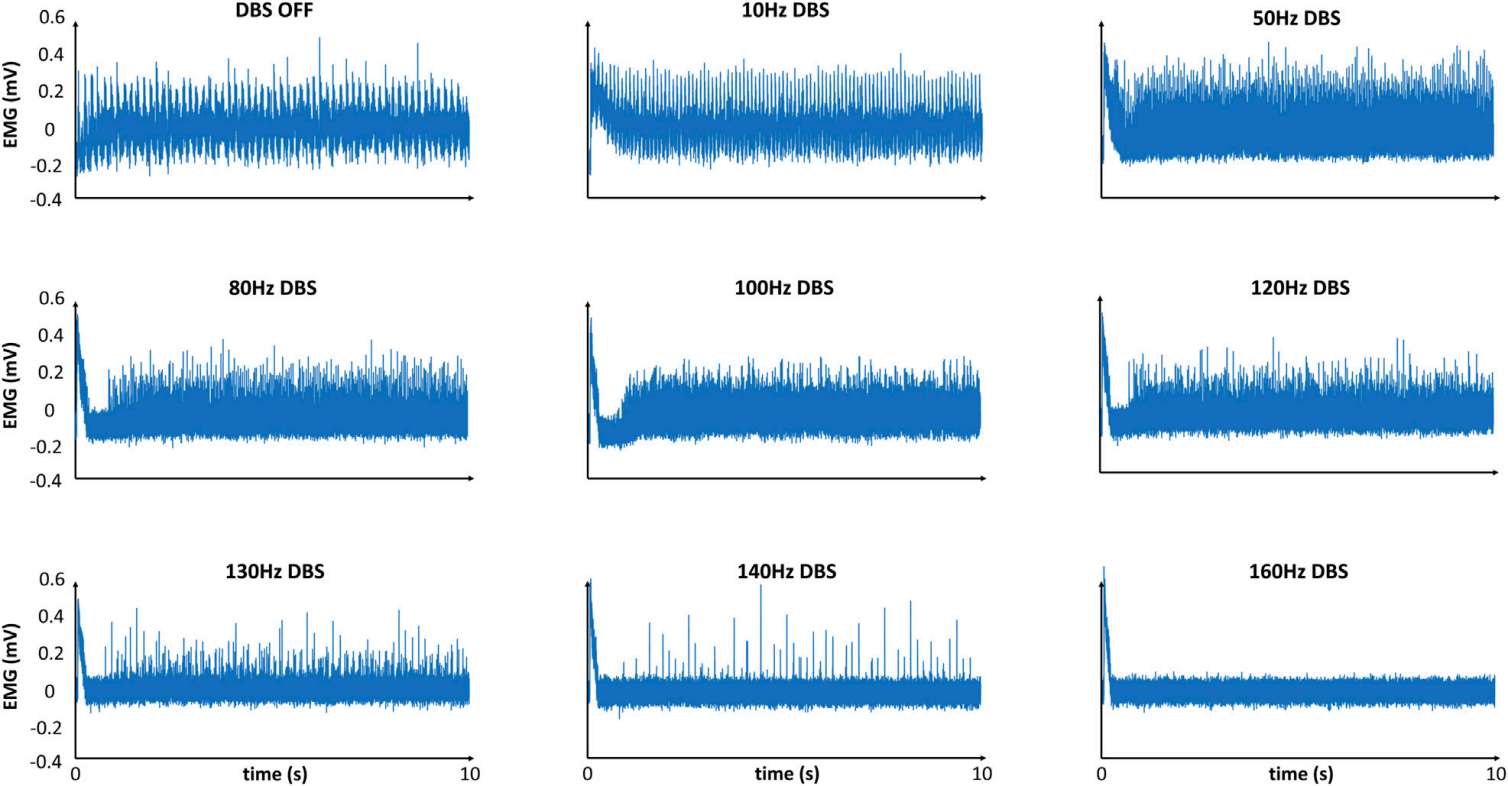
Model-simulated EMG in response to different frequencies of DBS. Simulated EMG with our model, in response to different frequencies of Vim–DBS. Each signal is given relative to its mean.

We simulated the EMG from our model, both during DBS-OFF and in response to Vim-DBS of different stimulation frequencies in [10–200] Hz (Figure 4).

The EMG simulation with DBS-OFF presented a typical tremor band (∼6 Hz) in the clinical EMG signals recorded from ET patients (Halliday et al., 2000; Hess and Pullman, 2012; Herron et al., 2017). During low-frequency ( ≤ 50 Hz) DBS, the amplitude of the simulated EMG is similar to (or slightly higher than) the DBS-OFF situation (Figure 4). Such a simulation is consistent with the clinical observations that low-frequency Vim-DBS ( ≤ 50 Hz) is often ineffective and can exacerbate the tremor (Ushe et al., 2004; Earhart et al., 2007; Pedrosa et al., 2013). The amplitude of the simulated EMG is lower than that of the DBS-OFF situation when the DBS frequency is ≥ 80 Hz (Figure 4). During high-frequency ( ≥ 100 Hz) DBS, the amplitude of the simulated EMG is clearly depressed compared with that in the DBS-OFF situation (Figure 4). Such a simulation is consistent with the clinical observations that high-frequency ( ≥ 100 Hz) Vim–DBS can worsen the tremor (Earhart et al., 2007; Ushe et al., 2004; Vaillancourt et al., 2003). The simulated EMG is mostly suppressed when the DBS frequency is ≥ 130 Hz (Figure 4). This is consistent with the fact that the stimulation frequency of clinical Vim–DBS is usually chosen to be ≥ 130 Hz (Ondo et al., 1998; Dowsey-Limousin, 2002; Dembek et al., 2020). We observed a short transient with a large amplitude in the simulated EMG during Vim–DBS, and the tremor intensity might be higher in the initial ∼200 ms after Vim–DBS onset (Milosevic et al., 2018; Yamamoto et al., 2013). Our simulated EMG signals are consistent with clinical EMG signals from Cernera et al. (2021), which showed EMG recordings from a patient with essential tremor during DBS-OFF and 135-Hz Vim-DBS (Cernera et al., 2021) (Supplementary Figure S6).

### Estimation of the simulated EMG

**FIGURE 5:**
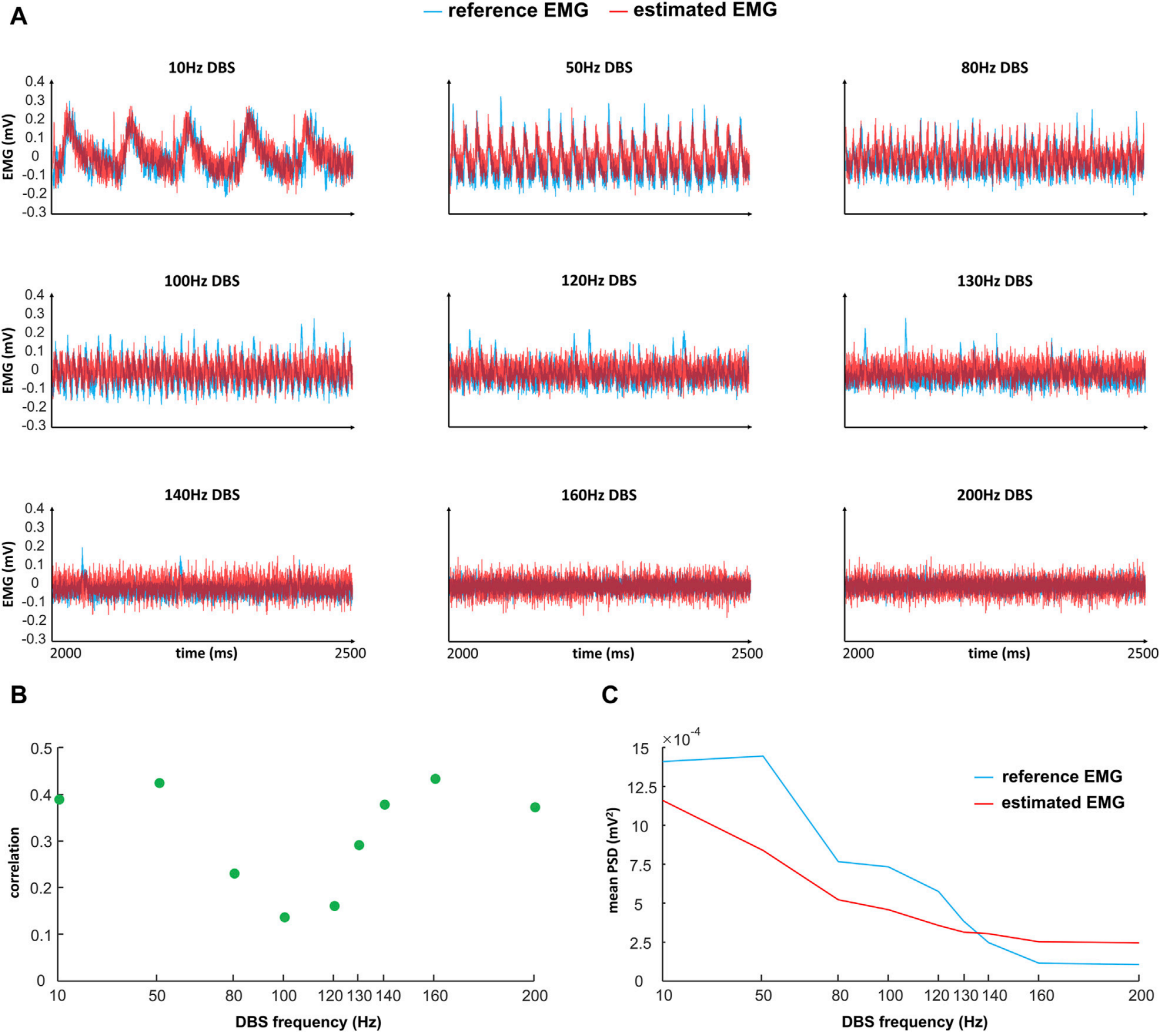
Comparison of reference and estimated EMGs (time domain) in response to different frequencies of DBS. “Reference EMG” is the EMG simulated by our model ( $y\left(t\right)$ in Figure 2). “Estimated EMG” is the estimation of the reference EMG with the polynomial fit ( $\hat{y}\left(t\right)$ in Figure 2). **(A)** Comparison of the reference EMG and estimated EMG in the time domain, in response to different frequencies of DBS. Each signal is subtracted by its mean. **(B)** Correlation between the reference EMG and estimated EMG. The correlation is computed based on the initial 5 s of data. **(C)** Comparison of the mean power spectral density (PSD) between the reference EMG and estimated EMG. Mean PSD is computed based on the initial 5 s of data.

**FIGURE 6:**
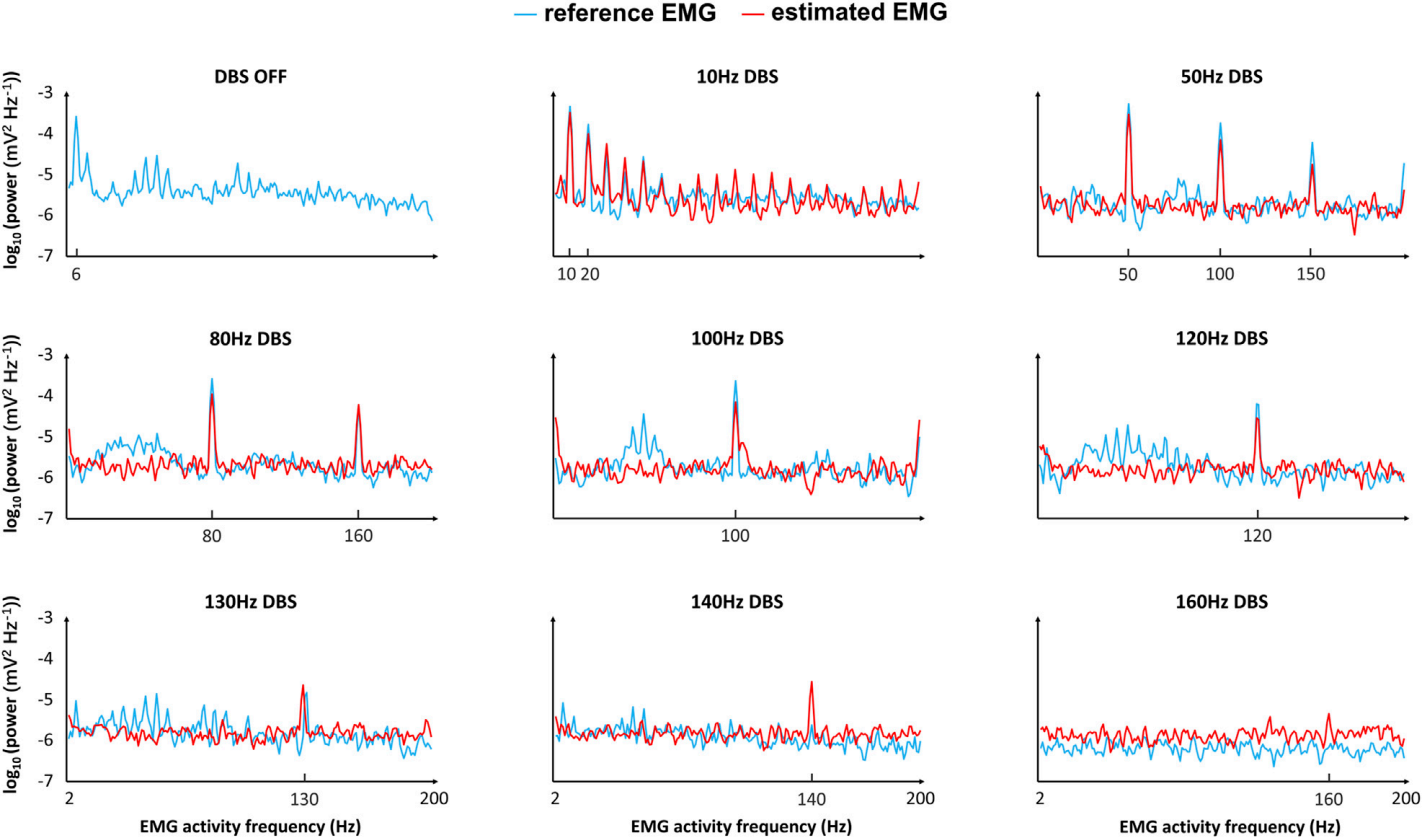
Comparison of the reference and estimated EMGs (frequency domain) in response to different frequencies of DBS. “Reference EMG” is the EMG simulated by our model ( $y\left(t\right)$ in Figure 2). “Estimated EMG” is the estimation of reference EMG with the polynomial fit ( $\hat{y}\left(t\right)$ in Figure 2). We compare the reference EMG and estimated EMG in the frequency domain, which is the frequency band [2–200] Hz of the EMG activities. For each DBS frequency, at each frequency of the EMG activities, we compute the corresponding frequency power with Eq. 8 in the initial T = 5 s of the EMG. The frequency power of EMG activities is plotted on a log scale.

The EMG simulation from the model is too slow for practical implementation. Thus, we estimated the model-simulated EMG with a polynomial fit to facilitate the computational speed in a closed-loop control system. The model-simulated EMG and polynomial-estimated EMG are denoted as “reference EMG” and “estimated EMG,” respectively (Figure 5). We compared the reference EMG and estimated EMG in response to different frequencies of DBS (Figures 5, 6).

In the time domain, the estimated EMG is similar to the reference EMG across different DBS frequencies (10–200 Hz), in terms of both amplitude and variation (Figure 5A). The correlation between the reference and estimated EMGs is generally above 0.3, representing some positive correlations (Figure 5B); the correlation is not very high because of the existence of white noise in the simulations. The power is generally similar between reference and estimated EMGs (Figure 5C). In addition to the comparison in the time domain, we also compared the reference and estimated EMGs in the frequency domain (Figure 6). At each frequency in the band [2–200] Hz of the EMG activities, we computed the corresponding frequency power with Eq. 8 in the initial T = 5 s of the EMG (Figure 6). The estimated EMG [by a 25-order polynomial (Eq. 6)] is well-fitted to the reference EMG in the frequency domain, with *R* ^2^ = 0.745 (Supplementary Figure S3), computed based on the signals across different DBS frequencies. Additionally, we observed other similarities between the estimated and reference EMGs in terms of the amplitude and pattern of different frequency powers of EMG activities (Figure 6). The EMG power is high with DBS-OFF and during low-frequency (<100 Hz) DBS and is mostly suppressed during ≥ 130 Hz DBS (Figure 6). During 10–80 Hz DBS, in both estimated and reference EMGs, we observed that the power is high at the harmonics of the DBS frequency (Figure 6). This might indicate that DBS could induce synchronized activities during low-frequency DBS (Florin et al., 2008; Pedrosa et al., 2013). The similarities between the reference and estimated EMGs—in both the time domain and frequency domain–indicate that the estimated EMG is a proper substitute for the reference EMG in controlling the frequencies of Vim–DBS for treating essential tremor.

### The system output based on EMG power spectral density

**FIGURE 7:**
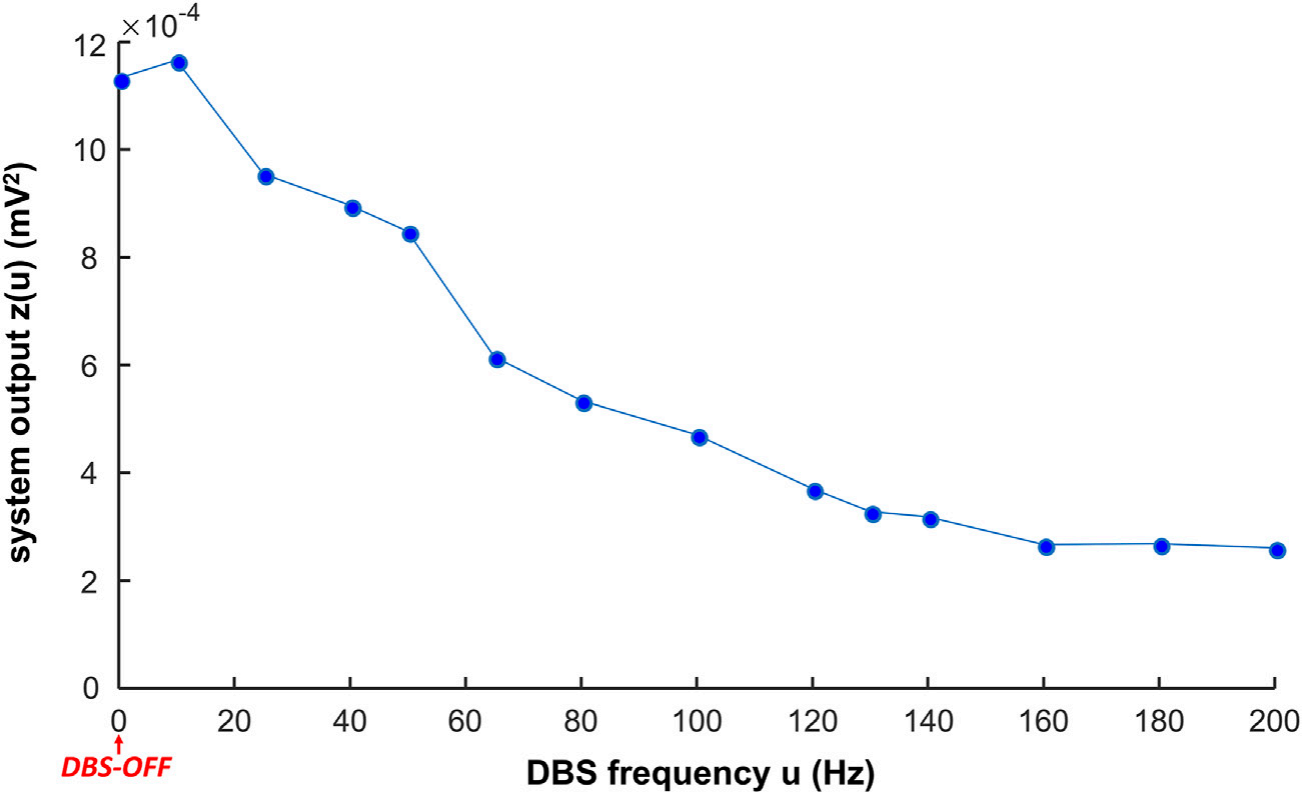
System output in response to different frequencies of DBS. The system output z(u) is the mean power spectral density (PSD) in the initial interval T = 5 s of the estimated EMG ( $\hat{y}\left(t\right)$ in Figure 2) in response to DBS with the simulation frequency u (Eq. 9).

We computed the system output to be implemented in a closed-loop controller updating the DBS frequency. The system output during Vim–DBS was computed with the estimated EMG (**Eq. 6**) that facilitates the implementation speed. For DBS frequency u , we defined the system output $z\left(u\right)$ as the mean power spectral density (PSD) in the initial interval T = 5 s of the estimated EMG in response to DBS with stimulation frequency u (Eq. 9). PSD represents the band [2–200] Hz of EMG activities. The system output in response to different frequencies ([10–200] Hz) of DBS is presented in Figure 7:

In general, the system output decreases with increase in the DBS frequency (Figure 7, Supplementary Table S3). During low-frequency ( ≤ 50 Hz) DBS, the system output is not reduced much compared with the DBS-OFF ( u = 0) situation (Figure 7). The system output is low during high-frequency ( ≥ 100 Hz) DBS and is close to minimum during ≥ 130 Hz DBS (Figure 7). These responses of the system output to different DBS frequencies are consistent with clinical observations of the effectiveness of different frequencies of Vim–DBS (Pedrosa et al., 2013; Earhart et al., 2007; Vaillancourt et al., 2003; Dembek et al., 2020).

### Closed-loop control of the DBS frequency with the PID controller

A PID controller (Eq. 10) was implemented to update the DBS frequency in the closed-loop system, based on the system output z (Eq. 9). The parameters $K_{p}$ , $K_{i}$ , and $K_{d}$ of the PID controller were chosen to be 10^3^ (Hz/mV^2^), 10^5^ Hz/(mV^2^ ∙ min), and 5 × 10^3^ (Hz ∙ min/mV^2^), respectively; parameter tuning was performed to increase the efficacy of the controller (Supplementary Figure S2 and Supplementary Note).

**FIGURE 8:**
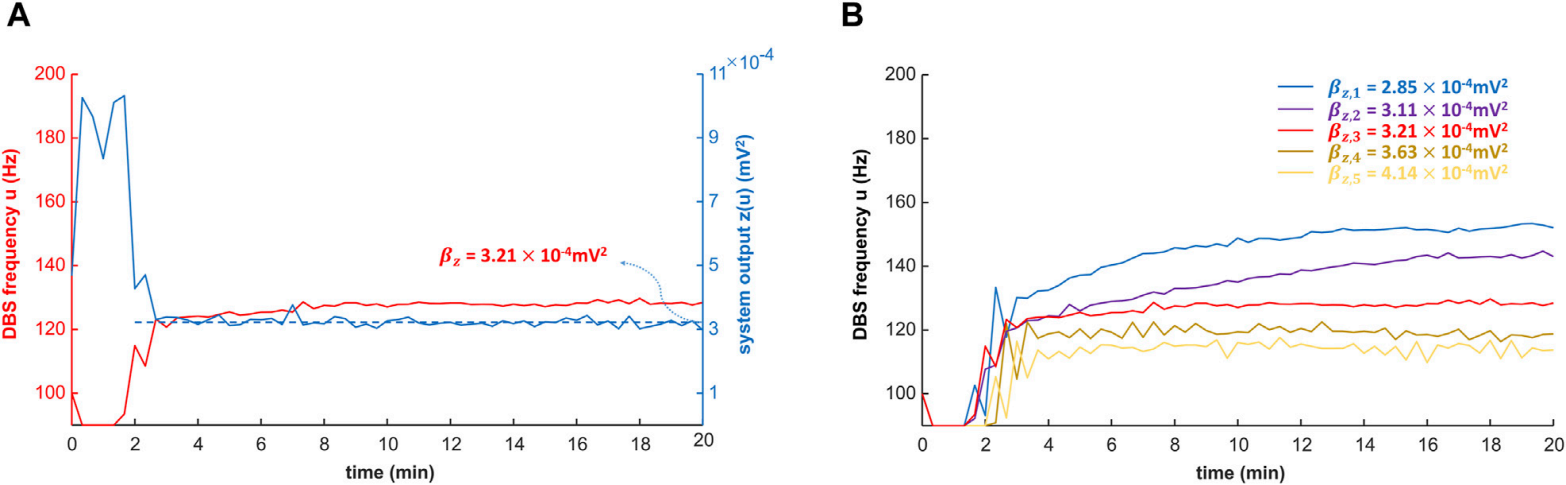
Closed-loop control of the DBS frequency with the PID controller. The DBS frequency is controlled in closed-loop with the proportional–integral–derivative (PID) controller (Eq. 10; Figure 2). The simulation of the PID controller is performed with MATLAB R2022b. **(A)** PID control of the DBS frequency with a specified target $\beta_{z}$ . During the control, the system output converges to the target, which is the power of the biomarker (estimated EMG, Eq. 6) simulated during 130-Hz DBS. **(B)** PID control of DBS frequency with different targets. $\beta_{z,1},\beta_{z,2},\ldots ,\beta_{z,5}$ represent five control targets of the system output z (Eq. 9 and Supplementary Table S4). $\beta_{z,2},\beta_{z,3}$ and $\beta_{z,4}$ correspond to the biomarker power during DBS frequency 140, 130, and 120 Hz, respectively. $\beta_{z,1}$ and $\beta_{z,5}$ correspond to unobserved DBS frequencies.

As a test of the controller, when the control target is the power of the biomarker (estimated EMG, Eq. 6) from 130-Hz DBS, the PID controller can converge to the target in 10 min (Figure 8A and Supplementary Table S4). This shows that our control system is potentially effective and efficient for clinical implementation. Note that during the PID control, only the steady-state DBS frequency (reached after ∼10 min) is delivered to the patient. As we change the control target of the system output, the result of the PID control is also robustly and flexibly changed (Figure 8B). As shown in Figure 8B, the five control targets of the system output correspond to the biomarker power from both observed and unobserved DBS frequencies (Supplementary Table S4). The observed DBS frequencies (10, 50, 80, 100, 120, 130, 140, 160, and 200 Hz) were used in fitting the polynomial coefficients (Eq. 6), and the unobserved DBS frequencies are arbitrary.

## Discussion

We developed a model-based closed-loop control system for the stimulation frequency of Vim–DBS. The DBS control system was based on our previously verified computational model, which represents the neuronal network characterizing the physiological mechanisms that connect the input (DBS pulses) and the output (model-predicted EMG activities). In order to facilitate the implementation speed, we estimated the model-predicted EMG with a polynomial fit, which was used as the feedback biomarker for the controller. The power spectrum of the biomarker was the system output implemented in a PID controller that automatically updates the appropriate DBS frequency. Thus, the closed-loop system controls the EMG power by adjusting the DBS frequency. Our closed-loop system can control the DBS frequency to achieve different control targets of EMG power and can potentially be implementable for different diseases and individual patients.

### Clinical relevance of the system output

The system output used in our closed-loop system is related to the power of the model-predicted EMG signals, and the optimal DBS frequency is obtained by bringing the system output to a specified control target. In clinical studies, the power of EMG is a commonly observed indicator for different movement disorders, e.g., PD (Zhang et al., 2017), ET (Halliday et al., 2000), and akinesia (Bisdorff et al., 1999). Tremor symptoms, characterized by the tremor amplitude and frequency, can be identified using the power of EMG (Hess and Pullman, 2012). Tremor amplitude is the primary indicator of the severity of tremors (Hess and Pullman, 2012). Tremor frequency can be used to partially differentiate disease types; e.g., the peak tremor frequency observed in the EMG of PD patients is often 3 ∼ 6 Hz (Zhang et al., 2017; Hess and Pullman, 2012) and in the EMG of ET patients is often 4 ∼ 8 Hz (Halliday et al., 2000; Herron et al., 2017). Thus, use of the power of EMG as a biomarker for ET in a closed-loop DBS is clinically relevant (Yamamoto et al., 2013; Herron et al., 2017). During the DBS control, the control target of the EMG power should be appropriate: a high EMG power indicates the insufficiency of tremor suppression, and a low EMG power can be related to akinesia (Bisdorff et al., 1999) and myasthenia gravis (Mills, 2005).

### Importance of predictability in a control system

The ability to predict how different frequencies of DBS change neural and behavioral activities is the main advantage of our model-based closed-loop DBS. A controller (e.g., PID) can select appropriate DBS patterns that are biophysically relevant and clinically effective. Although the model parameters [for both encoding and decoding models (see Figure 2) are obtained based on sparse DBS frequencies, 5, 10, 20, 30, 50, 100, 130, and 200 Hz [human Vim data (Tian et al., 2023b) and non-human primate cortical data (Agnesi et al., 2015)], the encoding model can robustly predict the effect of an arbitrary DBS frequency in the continuous spectrum of 5–200 Hz DBS (see Figure 4 for some examples; see Table 1 in Tian et al. (2023a) for a test of robustness of the firing rate model). Model-based control of DBS was addressed in previous computational studies (Fleming et al., 2020; Grado et al., 2018; Liu et al., 2021). Despite the usability of these control systems for *in silico* explorations, the underlying models were not fitted to experimental data. More importantly, these models do not consider the physiological mechanisms of the DBS effects. In this work, our (encoding) model not only incorporates biophysically realistic dynamics of DBS-induced short-term synaptic dynamics but also provides an accurate fit to Vim–DBS experimental data (Tian et al., 2023b). Additionally, our data-driven decoding model provides a fit (see Figures 5, 6) to simulated EMG data in which physiological mechanisms of tremor symptoms were preserved (see Figure 2 for details of the simulation study). Our control system predicts the effect of DBS frequency on the power of EMG and delivers an optimal DBS frequency.

### The use of machine learning methods in closed-loop DBS

In recent closed-loop DBS control systems, machine learning methods have been developed to map biomarker features (input) to patients’ observed states (output) and could further deliver an appropriate DBS setting (Chandrabhatla et al., 2023; Kuo et al., 2018; Merk et al., 2022). Therefore, it is imperative to identify key biomarkers that need to be extracted from the LFP and EMG as they will serve as input features for a judiciously selected machine learning model. Castaño-Candamil et al. (2020) used a regression method to estimate tremor severity from electrocorticographic (ECoG) power in ET patients and adjusted the DBS intensity according to tremor severity. Golshan et al. (2018) used the wavelet coefficients of the STN-LFP beta frequency range as features and further developed a support vector machine (SVM) classifier for studying the behaviors of PD patients. Numerous prior studies have established high performance using SVM classifiers with input features such as phase-amplitude coupling (Chandrabhatla et al., 2023), Hjorth parameters (Oliveira et al., 2023), beta band power (Chandrabhatla et al., 2023), and burst duration (Merk et al., 2022). Using power densities within the beta (Kuo et al., 2018) and gamma (Yao et al., 2020) bands as features, hidden Markov models (Merk et al., 2022; Sun et al., 2020; Yao et al., 2020), SVM (Golshan et al., 2018), convolutional neural networks (CNNs) (Merk et al., 2022; Golshan et al., 2020; Oliveira et al., 2023), linear discriminant analysis (LDA) (Merk et al., 2022), and logistic regression (Houston et al., 2019) have been investigated. It was also recommended in a couple of studies that deep learning methods such as CNNs are worth investigating as they capture nonlinear temporal dynamics and waveform shape (Merk et al., 2022; Oliveira et al., 2023). For example, Haddock et al. (2019) developed a deep learning method that classified the behaviors of ET patients based on the PSD of ECoG and used the classification results to turn DBS ON/OFF.

A key improvement to existing machine learning methods is to incorporate physiological characterizations of the input–output mapping. In this work, we developed a physiological model to map the input (DBS frequency) to the output (model-predicted EMG). We then used a polynomial-based approximation to estimate the input–output map to facilitate the implementation speed of the control system. However, the polynomial method is prone to be less robust to unseen inputs, owing to its high-order terms (Kane et al., 2017; Beltrão et al., 1991). Thus, an important line of future work is to use state-of-the-art machine learning methods, particularly deep learning methods, to replace the simple polynomial-based input–output mapping. Consequently, it is important to understand key EMG features for muscle activation in Parkinson’s disease. The literature highlights sample kurtosis, recurrence rate, and correlation dimension as three specific EMG features that are responsive to changes in DBS treatment parameters (Rissanen et al., 2015). These features have been fed as input into the LDA, CNN, and SVM, where the SVM performed the best (Ruonala, 2022). In a few investigations, EMG features, encompassing frequency, amplitude, and regularity, were scrutinized (Khobragade et al., 2018; Wang et al., 2020). The signal mean and power of the peak frequency performed well as features when using a random forest model and a deep learning network for adaptive DBS (Khobragade et al., 2018). It is important to note that while simpler models like LDA are valued (Ozturk et al., 2020; Watts et al., 2020) for their interpretability in the context of DBS for PD, the limited availability of labeled datasets resulted in ambiguous success for complex models, particularly deep neural networks (Oliveira et al., 2023).

Our closed-loop DBS control is based on a physiological model that can generate an arbitrary amount of synthetic data, which can be implemented in fully training deep learning methods. The arbitrary amount of data in the training set will increase the accuracy and robustness of our future closed-loop DBS control based on physiological models and deep learning methods. Therefore, it is recommended that the efficacy of the SVM, logistic regression, LDA, hidden Markov model (HMM), random forests, and deep neural network models like CNNs be evaluated in greater detail using the abundance of synthetic data. Watts et al. (2020) performed a thorough retroactive study of various machine learning classifiers used to identify optimal DBS parameters for PD, and it was recommended to pursue machine learning in the context of adaptive closed-loop DBS for PD.

### Different DBS mechanisms

Synaptic depression can partially explain the therapeutic mechanisms of high-frequency DBS (e.g., Vim–DBS), which can stem from synaptic and axonal failure (Rosenbaum et al., 2014; Tian et al., 2023a). In the present work, we incorporated dynamics of DBS-induced short-term synaptic plasticity (STP)—characterized by the Tsodyks and Markram model (Tsodyks et al., 1998)—in our neural model. Such a modeling strategy of the DBS effect is consistent with previous works (Farokhniaee and McIntyre, 2019; Milosevic et al., 2021). Further details of the DBS effect can also be considered in neuronal simulations. For example, Schmidt et al. (2020) modeled the effect of DBS by generating a spherical electrical field that affects the potential of all neuronal elements (including soma and axon) within a certain distance from the DBS electrode (Schmidt et al., 2020). The electrical field induced by DBS can be non-spherical if multiple electrodes or directional leads are used (Steffen et al., 2020; Masuda et al., 2022). DBS can induce both orthodromic and antidromic activations of axons, e.g., in Vim–DBS (Grill et al., 2008) and STN–DBS (Neumann et al., 2023). In particular, during STN–DBS, the antidromic activation of the cortical circuitry is a key factor in changing neural dynamics (Neumann et al., 2023). We will investigate the DBS effect of antidromic activations in our future models and compare different models of the DBS mechanisms.

### Limitations and future work

In our closed-loop system, the EMG power of a broad band is used as the system output to update the input DBS frequency. We used the band [2–200] Hz that covers the DBS frequencies [10–200] Hz, which induces DBS-evoked activities in our EMG simulations (Figures 5, 6). These DBS-evoked activities could be a mechanism of the ineffectiveness of low-frequency Vim–DBS (Ushe et al., 2004; Earhart et al., 2007; Pedrosa et al., 2013) and need to be suppressed in the closed-loop control system. Yet, EMG recordings during low-frequency DBS are very limited, and more such recordings are needed to fully investigate the underlying mechanisms. There have been closed-loop DBS systems controlling the tremor band [∼(2, 12) Hz] of EMG activities in ET patients (Cernera et al., 2021; Yamamoto et al., 2013). Cernera et al. (2021) showed that during DBS-OFF for an ET patient, most of the EMG power belongs to the band (2–12) Hz (Cernera et al., 2021). Thus, the control result may be similar when using the broad band [2–200] Hz, in which the power of [12–200] Hz is small, and the result will not be biased toward this relatively small power. In the future, we will perform the control of the tremor band ∼ (2–12) Hz of EMG activities and compare it with the current scheme.

We developed a model-based closed-loop control system that automatically updates the DBS frequency. Although the DBS frequency is a commonly tuned parameter in clinical applications (Merola et al., 2013), the tuning of another DBS parameter (e.g., pulse width and amplitude), or a combination of different DBS parameters, may also be clinically effective. Our closed-loop system adapts the DBS frequency because the underlying Vim network model was built to fit clinical data recorded during different frequencies of DBS. In the future, we will develop closed-loop systems that can adapt different DBS parameters, based on the corresponding new clinical data.

Our model-based closed-loop DBS control system is in the proof-of-concept stage for clinical implementations. The system will be implemented together with a constant monitoring of the EMG signal. In contrast to existing closed-loop DBS systems that update DBS parameters based on EMG signals solely (Yamamoto et al., 2013; Herron et al., 2017), before delivering DBS to the patient, our system predicts the effect of DBS frequency based on the underlying computational model. During implementation, the recorded EMG signal will be used to adjust our model predictions to personalize the model-based system and increase the prediction accuracy.

Our model included the clinical Vim–DBS data recorded in Vim neurons across different stimulation frequencies (10–200 Hz) (Tian et al., 2023b). However, experimental data were not sufficiently included in other components of our model. We incorporated non-human primate single-unit recordings in M1 during 130-Hz VPLo-DBS reported in Agnesi et al. (2015), but M1 activities in response to other DBS frequencies were not recorded. The EMG model simulation is consistent with some clinical observations, but was not further developed and validated by fitting clinical EMG data. In fact, in current experimental work on DBS, the EMG signal is usually recorded with DBS-OFF or high DBS frequencies (>100 Hz) (Herron et al., 2017; Vaillancourt et al., 2003; Rissanen et al., 2011), and they lack EMG recordings in response to a wide spectrum of DBS frequencies (e.g., 10–200 Hz). In the future, we plan to incorporate more experimental data into the further development of the model-based closed-loop DBS control, and these experimental data—in particular, cortical and EMG data—need to be recorded with different DBS frequencies from each individual subject.

It is worth mentioning that the synaptic connections between the Vim (VPLo in primate) are reciprocal and excitatory, though the projections that the M1 sends to the Vim are in different M1 laminae than the ones it receives from Vim (Stepniewska et al., 1994). Our model simplified this excitatory feedback relationship by considering the excitatory propagation effect as unidirectional, from the Vim to the M1. We fitted the simplified model to the recording from one M1 neuron during 130-Hz VPLo-DBS, and there are variabilities among the dynamics of different M1 neurons. In the future, we will develop a more detailed Vim-M1 model based on more M1 recordings. In this work, we modeled the spinal motoneurons as one population. Spinal motoneurons can be classified into two function groups: somatic and visceral (Fields et al., 1970). The M1–motoneuron synaptic projection can be modeled by pair-based STDP, which characterizes membrane potential dynamics using spike timings of both pre- and post-synaptic spikes (Morrison et al., 2008; Gütig et al., 2003). We will develop more detailed models of the M1–motoneuron circuits in the future. Our future closed-loop DBS systems will be constructed based on both improved models and deep learning methods.

## Data Availability

The raw data supporting the conclusion of this article will be made available by the authors, without undue reservation.
