# Supplementary material for: Model-based closed-loop control of thalamic deep brain stimulation
Source: Front Netw Physiol. 2024 Apr 8;4:1356653. doi: 10.3389/fnetp.2024.1356653 (PMC11033853; doi:10.3389/fnetp.2024.1356653)
Supplement: Supplementary file 1 [file DataSheet1.docx]

**Supplementary Method 1 – The Tsodyks & Markram Model of Short-Term Synaptic Plasticity**

The synapses of the neuron in the primary motor cortex (M1) were characterized by the Tsodyks & Markram (TM) model of short-term synaptic plasticity (STP) [1]. During Vim-DBS, we modeled that each M1 neuron receives DBS-induced inputs from 500 synapses, with 90% excitatory synapses ($N_{exc}$ = 450) and 10% inhibitory synapses ($N_{inh}$ = 50) ([2], **Table S1**). The DBS-induced inputs were from two Sources: (1) the direct DBS activation of the axons projected to the M1 neuron; and (2) the firings of the Vim neurons during Vim-DBS. In Source (1), we assumed that each DBS pulse generates a spike in each of the 500 synapses simultaneously [2][3]. In Source (2), from our recent Vim-network model [4], we simulated the instantaneous firing rate of the Vim neurons receiving DBS of different stimulation frequencies (10~200Hz). Then, the Vim firing rate signal was implemented as the time-varying Poisson rate to generate Poisson spike trains. The spikes from Sources (1) and (2) were passed to the TM model (**Equations 12-14**) to generate the DBS-induced post-synaptic current $I_{DBS}$.

Besides the DBS-induced inputs, the M1 neuron also received the background neuronal spikes that induce the tremor. We modeled the tremor-inducing background firing rate as a waveform consisting of 6-Hz bursts and a baseline shift, each burst consists of 3 consecutive sinusoidal waves, and the period of each wave is 20 ms (**Supplementary Figure 1**). We then generated Poisson spike trains from the background firing rate waveform, and these spikes were passed to TM model (**Equations** **12-14**) to generate the post-synaptic current $I_{b}$ induced by these background spikes generating the tremor. $I_{syn}= I_{DBS}+ I_{b}$ is the total post-synaptic input current that incorporates all the input spikes.

$I_{syn}$ was obtained by a linear combination of post-synaptic excitatory ($I_{exc}$) and inhibitory ($I_{inh}$) currents as follows:

$I_{syn}\left( t \right)= w_{exc} I_{exc}\left( t \right)- w_{inh}I_{inh}(t)$ (11)

where $w_{exc}$ and $w_{inh}$ denote the scaling weights of the excitatory and inhibitory currents, respectively (**Supplementary Table 1**). $I_{exc}$ (respectively, $I_{inh}$) is the total post-synaptic current from all excitatory (respectively, inhibitory) synapses; each synapse (excitatory or inhibitory) was modeled by the TM model for short-term synaptic plasticity:

$\frac{du}{dt}=-\frac{u}{\tau_{facil}}+U\left( 1-u^{-} \right)\delta\left( t-t_{sp} \right)$ (12) $\frac{dm}{dt}= \frac{1-m}{\tau_{rec}}-u^{+}m^{-}\delta\left( t-t_{sp} \right)$ (13)

$\frac{dI}{dt}=-\frac{I}{\tau_{s}}+Au^{+}r^{-}\delta(t-t_{sp})$ (14)

where $u$ is a utilization parameter, indicating the fraction of neurotransmitters ready for release into the synaptic cleft (due to calcium ion flux in the presynaptic terminal). The variable $m$ indicates the fraction of resources remaining available after the neurotransmitter depletion caused by neuronal spikes. We denote as $u^{-}$ and $m^{-}$ the corresponding variables just before the arrival of the spike; similarly, $u^{+}$ and $m^{+}$ refer to the moment just after the spike. The $\delta$–function models the abrupt change upon the arrival of each presynaptic spike $t_{sp}$; for example, at $t=t_{sp}$ in **Equation 12**, $u$ increases by $U\left( 1-u^{-} \right)$, and $\delta\left( t-t_{sp} \right)=$ 0 when $t\neq t_{sp}$. If there is no presynaptic activity (spike), $u$ exponentially decays to zero; this decay rate is the facilitation time constant, $\tau_{facil}$ (**Equation 12**). In contrast to the increase of $u$ upon the arrival of each presynaptic spike, $m$ drops and then recovers to its steady state value ($=$ 1); this recovery rate is given by the recovery time constant $\tau_{rec}$ (**Equation 13**). The competition between the facilitation ($\tau_{facil}$) and recovery ($\tau_{rec}$) time constants determined the dynamics of the synapse. In the TM model, $U$,$\tau_{facil}$, and $\tau_{rec}$ were the parameters that determine the three types of the synapse: facilitation (“F”), pseudo-linear (“P”), and depression (“S”) ([2], **Supplementary Table 1**). In **Equation 14**, $I$ is the post-synaptic current, $A$ is the absolute response amplitude, and $\tau_{s}$is the post-synaptic time constant (**Supplementary Table 1**). We obtained $I_{exc}$ (respectively, $I_{inh}$) by adding the post-synaptic currents from all excitatory (respectively, inhibitory) synapses. The TM model parameters in **Supplementary Table 1** were chosen based on the previous modeling works on specific experimental datasets [2][5][6].

**Supplementary Method 2 – Firing Rate Model of Vim-Network Impacted by Vim-DBS**

We developed a firing rate model of the Vim-network in patients with essential tremor (**Supplementary Figure 4**). The model was fitted to the experimental single-unit recordings of human Vim neurons receiving Vim-DBS with different frequencies – 5, 10, 20, 30, 50, 100, and 200Hz [2]. To optimize the model parameters, we developed a novel route optimization method to search for consistent model parameters that accurately replicate the experimental data across different DBS frequencies [4].

The rate network model of the Vim-DBS is illustrated in **Supplementary Figure 4**. The model consists of three differential equations, as stated below.

$\left\{ \begin{aligned} \tau_{e}\frac{dr_{D}}{dt}=-\left( r_{D}-r_{D,0} \right)+\left[ W\boldsymbol{r} \right]_{D}+F(I_{DBS}) \\ \tau_{e}\frac{dr_{E}}{dt}=-\left( r_{E}-r_{E,b} \right)+\left[ W\boldsymbol{r} \right]_{E} \\ \tau_{i}\frac{dr_{I}}{dt}=-\left( r_{I}-r_{I,0} \right)+\left[ W\boldsymbol{r} \right]_{I} \end{aligned} \right.$ (15)

where,

$\boldsymbol{r=}\left( \begin{aligned} r_{D} \\ r_{E} \\ r_{I} \end{aligned} \right)$,

$W=\left( \begin{matrix} w_{DD} & w_{DE} & w_{DI} \\ w_{ED} & w_{EE} & w_{EI} \\ w_{ID} & w_{IE} & w_{II} \end{matrix} \right)=\left( \begin{matrix} w_{ee} & w_{ee} & -w_{ei} \\ w_{ee} & w_{ee} & -w_{ei} \\ w_{ie} & w_{ie} & {-w}_{ii} \end{matrix} \right)$,

$F\left( I_{DBS} \right)=\frac{c}{1+exp[-s*\left( I_{DBS}-k \right)]}$ ;

*The parameters* $\Phi=\left\{ w_{ee}, w_{ie}, w_{ei}, w_{ii}, \tau_{i}, \tau_{e}, r_{E,b}, c, s, k \right\}$ *are undetermined*

In **Equation 15**, neural group “$D$” represents the Vim neurons directly receiving DBS, neural group “$E$” represents the external excitatory nuclei, and neural group “$I$” represents the external inhibitory nuclei (**Supplementary Figure 4**). The group “$E$” neurons are mainly the cerebellum dentate nucleus and the pyramidal cells of primary motor cortex (M1) deep layers (Layer 5 and 6) [7][2][8]. The group “$I$” neurons mostly consist of the thalamic reticular nucleus (TRN) and interneurons [9][2]. $r_{m}$ ($m\in\{D,E,I\}$) represents the firing rate of the corresponding neural group. The baseline firing rate (with DBS–OFF) of Vim neurons ($r_{D,0}$) and the average firing rate of external inhibitory nuclei ($r_{I,0}$) were obtained from single-unit recordings reported in other studies [2][10]. We chose $r_{D,0}$ = 25 Hz to be consistent with the human Vim experimental recordings in our previous work Milosevic et al. (2021) [2], and $r_{I,0}$ = 5 Hz to be consistent with the experimental data recorded in both TRN (human) [2] and thalamic interneurons (mice) [10]. Since the external excitatory nuclei (group “$E$”) originate from multiple sources with highly variable firing rates [11][12], the corresponding baseline firing rate ($r_{E,b}$) is left as an unknown variable (**Equation 15**). In the experimental recordings from mice, the regular firing rate of cerebellum dentate nucleus ranged from 10 to 80 Hz [11][13]. For M1 Layer 5 and 6 neurons in mice, the firing rate ranged from 10 to 60 Hz [12][14]. Thus, we constrained $r_{E,b}$ in [10,70] Hz, and initialize it at $r_{E,b,0}$= 40 Hz.

In the rate network model, we use $\tau_{e}$ and $\tau_{i}$ to denote the excitatory and inhibitory time constants, respectively (**Equation 15**). Since Vim neurons are excitatory [2], the time constant of the neural group “$D$” was $\tau_{e}$. In a firing rate model of a population of neurons, the time constant ($\tau$) represents the changing speed of the firing rate in response to the post-synaptic current [15]. Generally, time constants in firing rate models were considered in the range of 0 to 30 ms [15][16]. The rate model time constant is consistent with the membrane time constant [15], and generally, the time constant of the inhibitory neurons is larger than that of the excitatory neurons [2][17].

The matrix W (**Equation 15**) indicates strength of connectivity between different groups of neurons [18][19]. In matrix W, $w_{pq}$ (for $p$,$q$ belong to group “$D$”, “$E$”, “$I$”) represents the connectivity strength from the neural group “$q$” to group “$p$”, and the +/- sign denotes the excitatory/inhibitory effect. The total network input into each neural group is computed as the matrix multiplication $W\boldsymbol{r=}{\boldsymbol{(}\left[ W\boldsymbol{r} \right]_{D}, \left[ W\boldsymbol{r} \right]_{E}, \left[ W\boldsymbol{r} \right]_{I})}^{T}$, where $\left[ W\boldsymbol{r} \right]_{D}$, $\left[ W\boldsymbol{r} \right]_{E}$ and $\left[ W\boldsymbol{r} \right]_{I}$ represent the inputs into group “$D$”, “$E$” and “$I$”, respectively (**Equation 15**).

For the Vim neurons directly receiving DBS (group “$D$”), we modeled the DBS-induced post-synaptic current ($I_{DBS}$) with the Tsodyks & Markram model [1] of short-term synaptic plasticity (STP) in agreement with Milosevic et al. (2021) [2]; $I_{DBS}$ is then transformed (with a sigmoid function) to the corresponding firing rate dynamics $F\left( I_{DBS} \right)$ ([3], **Supplementary Figure 4**, **Equation 15**). In the sigmoid transfer function $F\left( I_{DBS} \right)$, $c$, $s$ and $k$ are the scale, shape and shift parameters, respectively (**Equation 15**).

The undetermined parameter set in the rate network model is $\Phi=\left\{ w_{ee}, w_{ie}, w_{ei}, w_{ii}, \tau_{i}, \tau_{e}, r_{E,b}, c, s, k \right\}$ (see **Equation 15**). All rate model simulations were conducted with the sampling frequency at 10^4^ Hz.

The model parameters in the rate network model (**Equation 15**) were estimated based on the experimental single-unit recordings of human Vim neurons in patients undergoing DBS surgery for essential tremor. While recording activity of an individual Vim neuron, DBS was applied with one of the stimulation frequencies, {5, 10, 20, 30, 50, 100, and 200 Hz}, with specific stimulation length {10, 5, 3, 2, 1, 5, and 2 s}, respectively [2][4]. We obtained 5~8 recordings during each frequency of DBS, and computed the instantaneous firing rate with a time histogram method based on these multiple recordings [4]. The instantaneous firing rate was computed by convolving the experimentally recorded spike trains with an optimized Gaussian kernel that best characterized the spikes using a Poisson process [20][21][4]. We concatenated the instantaneous firing rate of each DBS frequency, and optimized the consistent model parameters across different DBS frequencies (5 to 200Hz) [4][3]. In **Supplementary Figure 5**, we showed the instantaneous firing rate calculated from experimental data, the results of our model fit, and the optimal model parameters.

As shown in **Supplementary Figure 5**, the rate network model accurately reproduced the recorded firing rates across different DBS frequencies (5 to 200Hz). The model could capture both transient and steady-state firing rate responses to each frequency of DBS. In particular, for 100-Hz and 200-Hz DBS data, the model is almost identical to the experimental data; this improves the result from our previous model that incorporated a single population of Vim neurons, and ignored the recurrent connections with other nuclei [3]. For Vim-DBS, high-frequency DBS (100 to 200Hz) is more clinically effective than low-frequency DBS (<100Hz) [22][23]. Additionally, we optimized the model parameters based on the concatenated signal across different DBS frequencies (5 to 200Hz). When fitting a rate model to such DBS data with concatenated frequencies, the fit accuracy is consistent between observed and unobserved DBS frequencies (e.g., 130Hz, 160Hz; see Table 1 in Tian et al. (2023) [3]).

We quantitatively validated the model goodness of fit by computing the normalized mean squared error (NMSE) between the experimental instantaneous firing rate (reference) and the model generated firing rate. Since high-frequency (100 to 200Hz) Vim-DBS is more clinically effective [22][23], we emphasized the high-frequency DBS data, and defined the total fitting error (ER) for model validation:

$ER = \frac{1}{3} * NMSE (5 to 50Hz) + \frac{1}{3}* NMSE (100Hz) + \frac{1}{3}* NMSE (200Hz)$ (16)

The NMSE(5 to 50Hz) represents the NMSE of the model fit to the concatenated data from DBS frequencies 5 to 50Hz. NMSE(100Hz) and NMSE(200Hz) were computed with data from the 100Hz and 200Hz DBS, respectively. For the rate network model fit shown in **Supplementary Figure 5**, ER = 7.6%, with NMSE(5 to 50Hz) = 13.9%, NMSE(100Hz) = 3.2% and NMSE(200Hz) = 5.7%. The optimal model parameters (**Supplementary Figure 5**) were obtained with our route optimization method [4].

**Supplementary Note – Parameter Tuning of the PID Controller**

We manually tuned the parameters ($K_{p}$, $K_{i}$, $K_{d}$) of the PID controller. The purpose of the PID parameter tuning was to increase the control efficacy. The parameters $K_{p}$, $K_{i}$, and $K_{d}$ were chosen to be 10^3^ (Hz/mV^2^), 10^5^ Hz/(mV^2^$\cdot$min), and 5$\times$10^3^ (Hz$\cdot$min/mV^2^), respectively. For each of the three PID parameter, we compared our choice with other values in a control experiment (i.e., the other two parameters are the same as our choices) (**Supplementary Figure 2**). Our PID parameter choices are compared with other values in the situation where the target DBS frequency is 130Hz (**Supplementary Figure 2A**). The results from the PID controller were constrained to be in the DBS frequency range [10, 200] Hz, and this wide frequency range contributes to the full investigation of the behavior of the PID controllers.

In the tuning of $K_{p}$ (**Supplementary Figure 2B**), we observed that the results are non-robust when $K_{p}\leq$10^2^ or $K_{p}\geq$5$\times$10^4^. The results with $K_{p}$ = 10^4^ is similar to our choice ($K_{p}$ = 10^3^), but the predicted DBS frequency is less accurate (**Supplementary Table 5**). In the tuning of $K_{i}$ (**Supplementary Figure 2C**), we observed that the results are not accurate when $K_{i}\geq$2$\times$10^5^ (the controller converges to a wrong value), and not efficient when $K_{i}\leq$5$\times$10^4^ (the controller takes a longer time to converge). Our choice ($K_{i}$ = 10^5^) is more accurate and efficient than the other shown values. In the tuning of $K_{d}$ (**Supplementary Figure 2D**), we observed that the result with our choice ($K_{d}$ = 5$\times$10^3^) is more robust than the results with other shown values.

**Supplementary Tables**

| ***excitatory synapses***  $\boldsymbol{N}_{\boldsymbol{exc}}$ **= 450;** $\boldsymbol{w}_{\boldsymbol{exc}}$ **= 49.00** | | | | | | ***inhibitory synapses***  $\boldsymbol{N}_{\boldsymbol{inh}}$ **= 50;** $\boldsymbol{w}_{\boldsymbol{inh}}$ **= 73.08** | | | | | |
| --- | --- | --- | --- | --- | --- | --- | --- | --- | --- | --- | --- |
| **para**    **type** | $U$ | $\tau_{facil}$  (ms) | $\tau_{rec}$  (ms) | $\tau_{s}$  (ms) | $A$ | **para**    **type** | $U$ | $\tau_{facil}$  (ms) | $\tau_{rec}$  (ms) | $\tau_{s}$  (ms) | $A$ |
| **F (40%)** | 0.19 | 670 | 138 | 2 | 1 | **F (40%)** | 0.016 | 376 | 45 | 8.5 | 1 |
| **P (20%)** | 0.45 | 326 | 329 |  |  | **P (30%)** | 0.29 | 62 | 144 |  |  |
| **S (40%)** | 0.04 | 17 | 85 |  |  | **S (30%)** | 0.25 | 21 | 706 |  |  |

**Supplementary Table 1. Tsodyks & Markram Model Parameters for Synapses Projected to one M1 Neuron**

*We show the parameters related to the Tsodyks & Markram model* [1]*, which is implemented to compute the post-synaptic current (*$I_{syn}$ *,* ***Equation 11****) into the neuron in the primary motor cortex (M1). For one M1 neuron, we model that it receives inputs from 500 synapses, with 90% excitatory synapses (*$N_{exc}$ *= 450) and 10% inhibitory synapses (*$N_{inh}$ *= 50).* $w_{exc}$ *and* $w_{inh}$ *are the scaling weights of the post-synaptic excitatory (*$I_{exc}$*) and inhibitory (*$I_{inh}$*) currents (****Equation 11****). Both excitatory and inhibitory synapses consist of 3 types: facilitation (“F”), pseudo-linear (“P”), and depression (“S”). For excitatory synapses, “F (40%)” represents “the facilitation type of synapses account for 40% of all the excitatory synapses”; similar meanings for other synaptic types, and the inhibitory synapses. “para” represents “the Tsodyks & Markram model parameters”, which consist of “*$U$*” (scaling factor), “*$\tau_{facil}$*” (facilitation time constant), “*$\tau_{rec}$*” (recovery time constant), “*$\tau_{s}$*” (post-synaptic time constant) and “*$A$*” (absolute response amplitude) (****Equations 2 – 4****).*

| $\varphi_{0}$ | 0.119 |
| --- | --- |
| $\varphi_{1}$ | 0.0891 |
| $\varphi_{2}$ | 2.12 |
| $\varphi_{3}$ | 4.26 |
| $\varphi_{4}$ | -21.3 |
| $\varphi_{5}$ | -29.0 |
| $\varphi_{6}$ | 97.8 |
| $\varphi_{7}$ | 61.0 |
| $\varphi_{8}$ | -256 |
| $\varphi_{9}$ | 7.15 |
| $\varphi_{10}$ | 369 |
| $\varphi_{11}$ | -208 |
| $\varphi_{12}$ | -230 |
| $\varphi_{13}$ | 295 |
| $\varphi_{14}$ | -31.3 |
| $\varphi_{15}$ | -136 |
| $\varphi_{16}$ | 97.7 |
| $\varphi_{17}$ | -12.7 |
| $\varphi_{18}$ | -20.7 |
| $\varphi_{19}$ | 16.2 |
| $\varphi_{20}$ | -6.37 |
| $\varphi_{21}$ | 1.59 |
| $\varphi_{22}$ | -0.264 |
| $\varphi_{23}$ | 0.0284 |
| $\varphi_{24}$ | -1.80$\times$10^-3^ |
| $\varphi_{25}$ | 5.16$\times$10^-5^ |

**Supplementary Table 2. Coefficients of the Polynomial Fit (Equation 5) (3 significant digits)**

| **DBS frequency u (Hz)** | **system output z(u) (10^-4^mV^2^)** |
| --- | --- |
| 0 | 11.31 |
| 10 | 11.66 |
| 25 | 9.53 |
| 40 | 8.94 |
| 50 | 8.45 |
| 65 | 6.10 |
| 80 | 5.29 |
| 100 | 4.65 |
| 120 | 3.63 |
| 130 | 3.21 |
| 140 | 3.11 |
| 160 | 2.59 |
| 180 | 2.60 |
| 200 | 2.53 |

**Supplementary Table 3. System output in Response to Different Frequencies of DBS**

*The system output z(u) is the mean power spectral density (PSD) in the initial T = 5s of the estimated EMG (*$\hat{y}(t)$ *in* ***Figure 2****) in response to DBS with stimulation frequency = u (Hz) (****Equation 9****).*

| **time**  **target** | **5min** | **10min** | **15min** | **20min** |
| --- | --- | --- | --- | --- |
| $\boldsymbol{\beta}_{\boldsymbol{z}\boldsymbol{,}\boldsymbol{1}}$ | 137.2Hz | 148.8Hz | 151.8Hz | 152.0Hz |
| $\boldsymbol{\beta}_{\boldsymbol{z}\boldsymbol{,}\boldsymbol{2}}$ **= z(140)** | 126.0Hz | 135.0Hz | 141.7Hz | 143.0Hz |
| $\boldsymbol{\beta}_{\boldsymbol{z}\boldsymbol{,}\boldsymbol{3}}$ **= z(130)** | 125.5Hz | 127.1Hz | 127.1Hz | 128.4Hz |
| $\boldsymbol{\beta}_{\boldsymbol{z}\boldsymbol{,}\boldsymbol{4}}$ **=z(120)** | 120.4Hz | 119.4Hz | 117.1Hz | 118.8Hz |
| $\boldsymbol{\beta}_{\boldsymbol{z}\boldsymbol{,}\boldsymbol{5}}$ | 114.7Hz | 116.1Hz | 114.9Hz | 113.8Hz |

**Supplementary Table 4. Updated DBS Frequency during PID Control with Different Targets**

*“PID” represents the proportional-integral-derivative controller (****Equation 10****).* $\beta_{z,1},\beta_{z,2}, \ldots, \beta_{z,5}$ *represent 5 target values of the system output z (****Figure 9B*** *and* ***Equation 9****). The values of* $\beta_{z,1},\beta_{z,2}, \ldots, \beta_{z,5}$ *were specified in the legend of* ***Figure 9B****. This table shows the updated DBS frequency in the process of the PID control, with different target values of the system output.* $\beta_{z,2}$*,* $\beta_{z,3}$*, and* $\beta_{z,4}$ *are system output target values with target DBS frequency 140Hz, 130Hz and 120Hz, respectively (****Supplementary Table 3****).* $\beta_{z,1}$ *and* $\beta_{z,5}$ *are system output target values corresponding to unknown DBS frequencies, to be explored by the PID controller.*

| **time**  $\boldsymbol{K}_{\boldsymbol{p}}$ | **5min** | **10min** | **15min** | **20min** |
| --- | --- | --- | --- | --- |
| **10^2^** | 200.0Hz | 187.2Hz | 167.9Hz | 149.4Hz |
| **10^3^ (selected)** | 122.7Hz | 127.7Hz | 129.4Hz | 130.2Hz |
| **10^4^** | 121.0Hz | 125.3Hz | 126.6Hz | 128.0Hz |
| **5**$\boldsymbol{\times}$**10^4^** | 127.3Hz | 126.1Hz | 129.7Hz | 126.7Hz |
| **10^5^** | 124.2Hz | 121.6Hz | 123.6Hz | 127.2Hz |

**Supplementary Table 5. Updated DBS Frequency during PID Control with Different** $\boldsymbol{K}_{\boldsymbol{p}}$ **Values**

*“PID” represents the proportional-integral-derivative controller, and* $K_{p}$ *denotes the proportional gain of the PID controller (****Equation 10****). This table shows the updated DBS frequency in the process of the PID control with target DBS frequency 130Hz, using different values of* $K_{p}$*. The corresponding PID control processes are presented in* ***Supplementary Figure 2B****. The selected* $K_{p}$ *value is 10^3^ (****Supplementary Figure 2A****).*


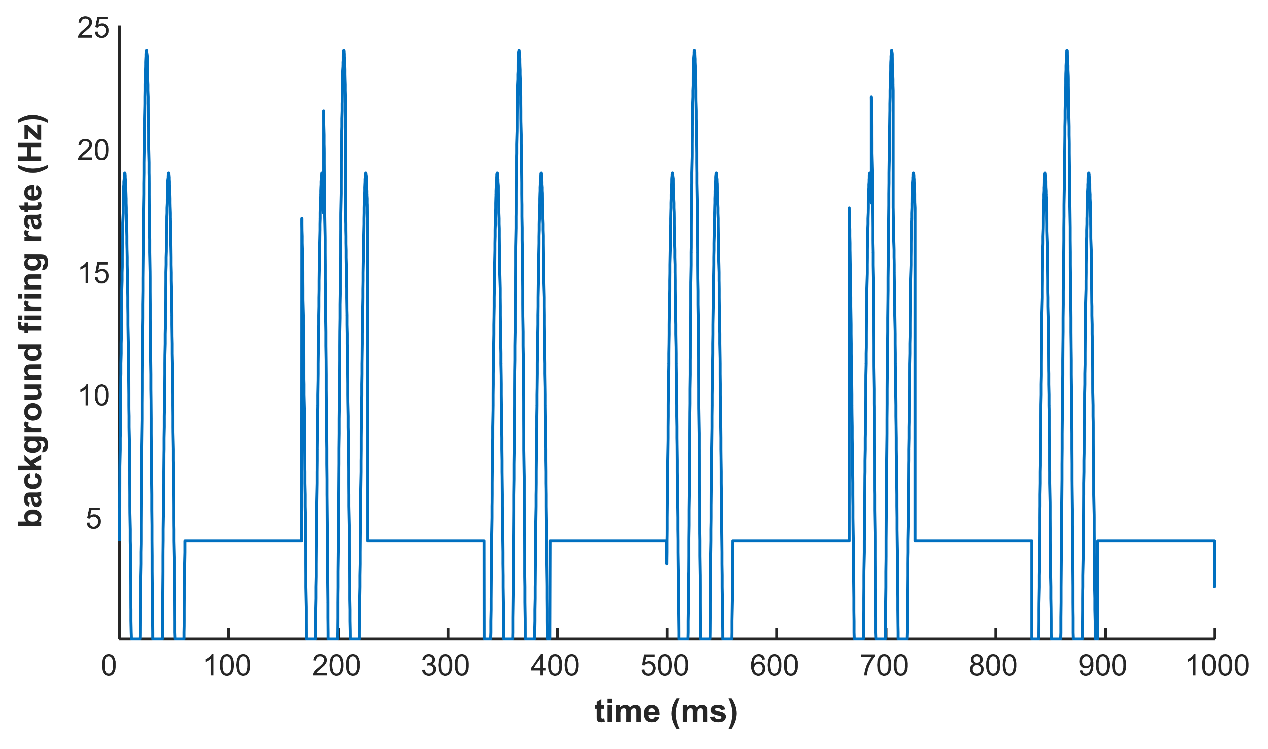


**Supplementary Figure 1. Background Cortical Inputs that Induce Essential Tremor**

*The background inputs into the neuron in the primary motor cortex (M1) induce essential tremor symptoms, which are often in the frequency band 4~8Hz. We model these background inputs as a firing rate waveform consisting of 6Hz bursts and a baseline shift. Each burst consists of 3 consecutive sinusoidal waves and the period of each wave is 20ms.*

**
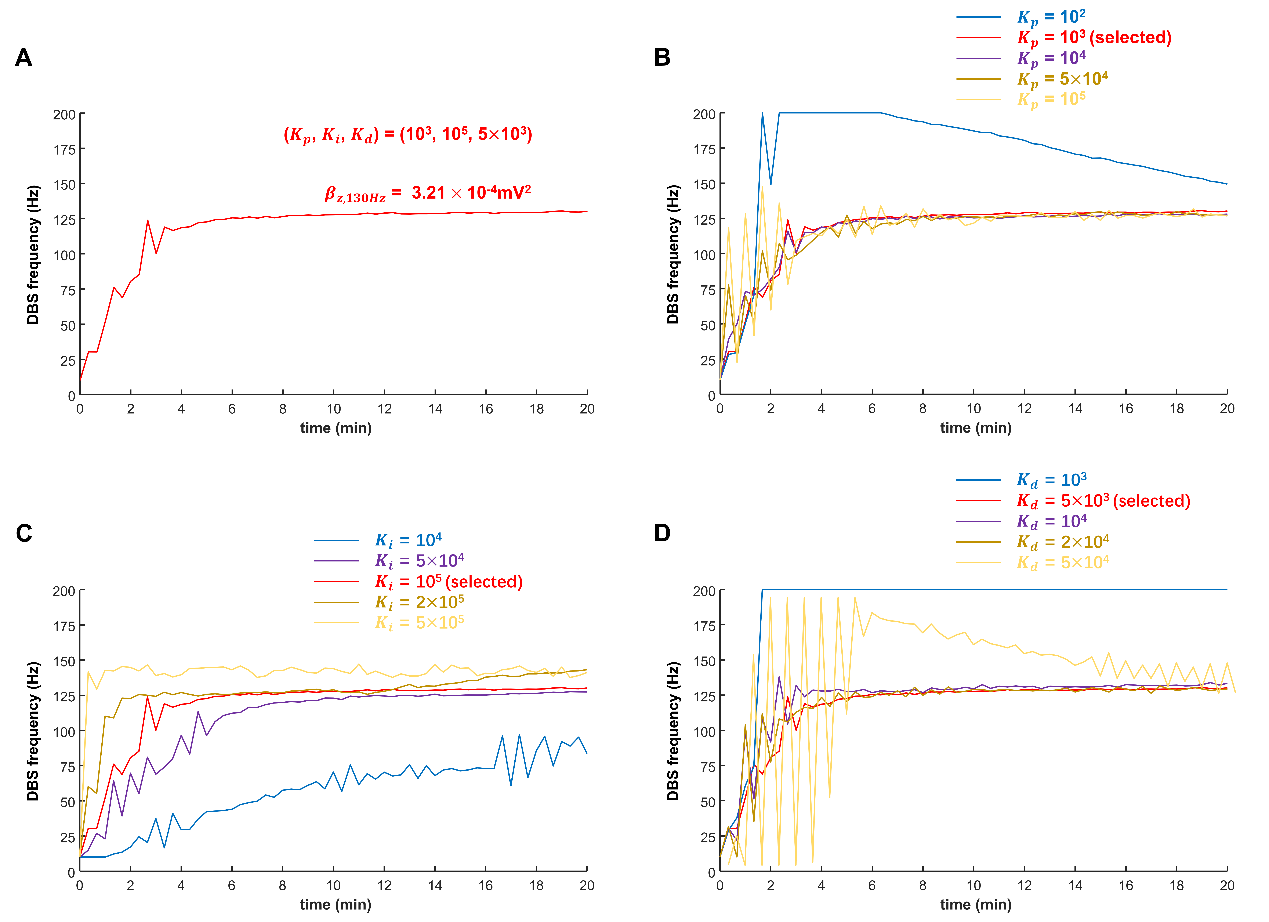
**

**Supplementary Figure 2. Parameter Tuning of the PID Controller**

*We compare the impact of different parameters (*$K_{p}$*,* $K_{i}$*,* $K_{d}$*) of the proportional-integral-derivative (PID) controller (****Equation 10****). We test the parameters (*$K_{p}$*,* $K_{i}$*,* $K_{d}$*) of PID control with the target DBS frequency 130 Hz. The results from the PID control are constrained to be in the DBS frequency range [10, 200] Hz.*

*(A) Our choice of the parameters (*$K_{p}$*,* $K_{i}$*,* $K_{d}$*), and the PID control process corresponding to these selected parameters.* $\beta_{z,130Hz}$ *is the value of the system output z (****Equation 9****) corresponding to DBS frequency = 130 Hz.*

*(B) The PID control processes corresponding to different* $K_{p}$*. The other two parameters (*$K_{i}$*,* $K_{d}$*) are the same as our decisions in (A), in all the PID control processes.*

*(C) The PID control processes corresponding to different* $K_{i}$*. The other two parameters (*$K_{p}$*,* $K_{d}$*) are the same as our decisions in (A), in all the PID control processes.*

*(C) The PID control processes corresponding to different* $K_{d}$*. The other two parameters (*$K_{p}$*,* $K_{i}$*) are the same as our decisions in (A), in all the PID control processes.*

*
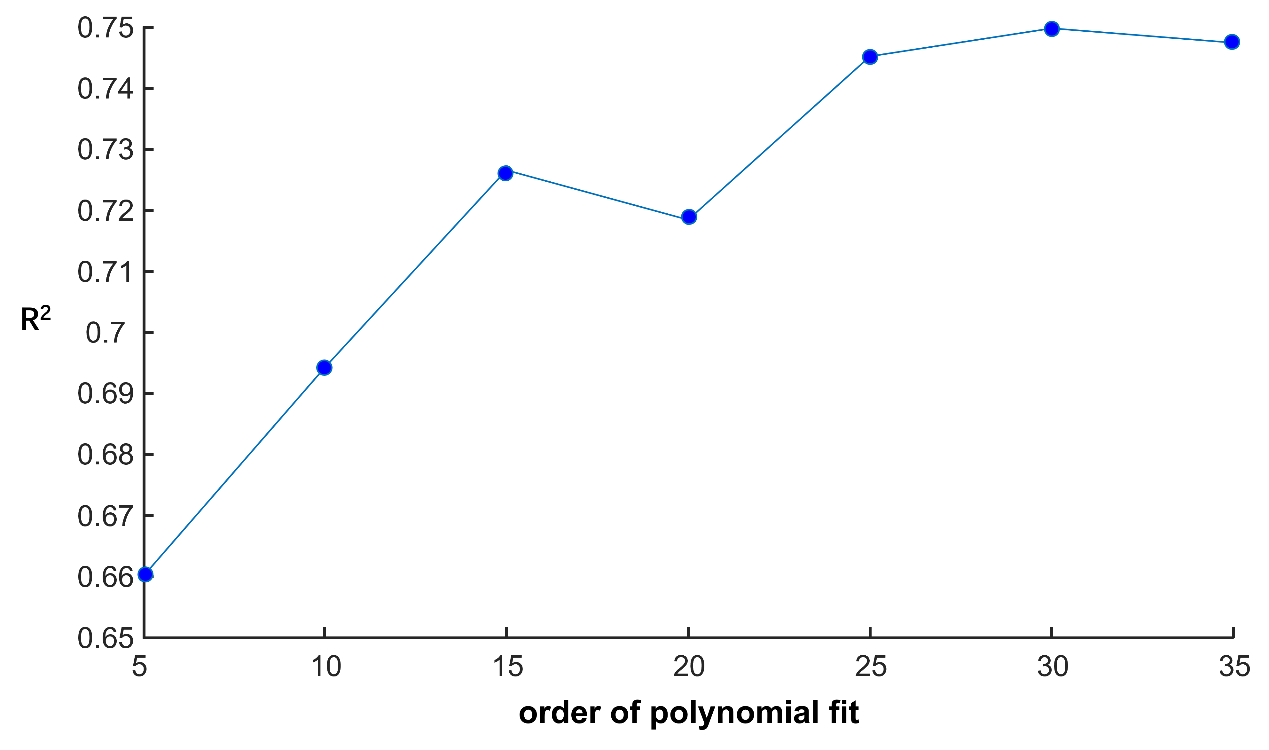
*

**Supplementary Figure 3. Comparison of** $\boldsymbol{R}^{\boldsymbol{2}}$ **of Different Orders of Polynomial Fits Using Frequency Domain Signals**

*We compare the* $R^{2}$ *statistic of different orders of polynomial fits using the corresponding frequency domain signals across different DBS frequencies (illustrated in* ***Figure 7****). The data length is 10s for each DBS frequency in {10, 50, 80, 100, 120, 130, 140, 160, and 200Hz}.*


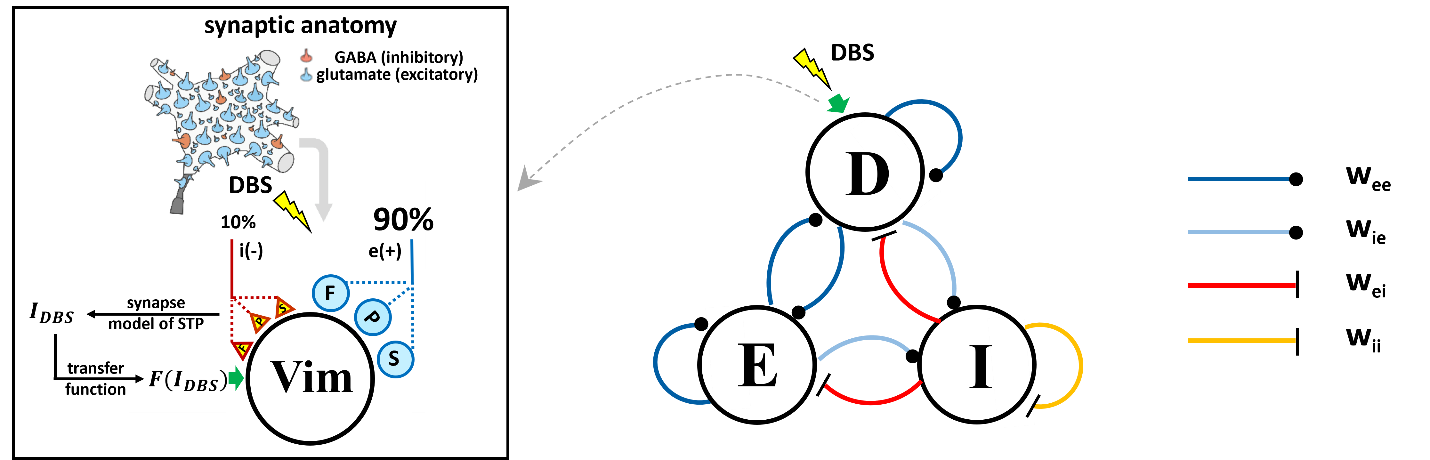


**Supplementary Figure 4**. **Schematic illustration of the rate network model**

*The firing rate network model consists of 3 recurrent neural groups: “*$D$*” represents the ventral intermediate nucleus (Vim) neurons directly receiving DBS, “*$E$*” represents the external excitatory nuclei, and “*$I$*” represents the external inhibitory nuclei.* $w_{ei}$ *represents the connectivity strength from inhibitory neurons to excitatory neurons; similar meanings for* $w_{ee}$*,* $w_{ie}$ *and* $w_{ii}$*. The connection with a dot (respectively, a bar) represents excitation (respectively, inhibition). DBS is delivered to the neural group* $D$*, and we analyze the synaptic anatomic structure within group* $D$*, which consists of Vim neurons. A DBS pulse simultaneously activates all the synapses projecting to a Vim neuron in group* $D$*; 90% of these synapses are excitatory, and 10% of these synapses are inhibitory* [2]*. An excitatory synapse mainly projects glutamate neurotransmitter, and an inhibitory synapse mainly projects gamma-aminobutyric acid (GABA) neurotransmitter* [2]*. Excitatory (respectively, inhibitory) synapses consist of 3 types: “F” (facilitation), “P” (pseudo-linear)”, and “S” (depression)* [2]*. We formulate the DBS-induced post-synaptic current (*$I_{DBS}$*) with the Tsodyks & Markram model of short-term synaptic plasticity (STP) (*[1]*,* [4]*);* $I_{DBS}$ *is then transferred (with a sigmoid function) to the corresponding firing rate dynamics* $F\left( I_{DBS} \right)$ *(****Equation 15****).* $F\left( I_{DBS} \right)$ *is the external DBS input into the rate network model.*


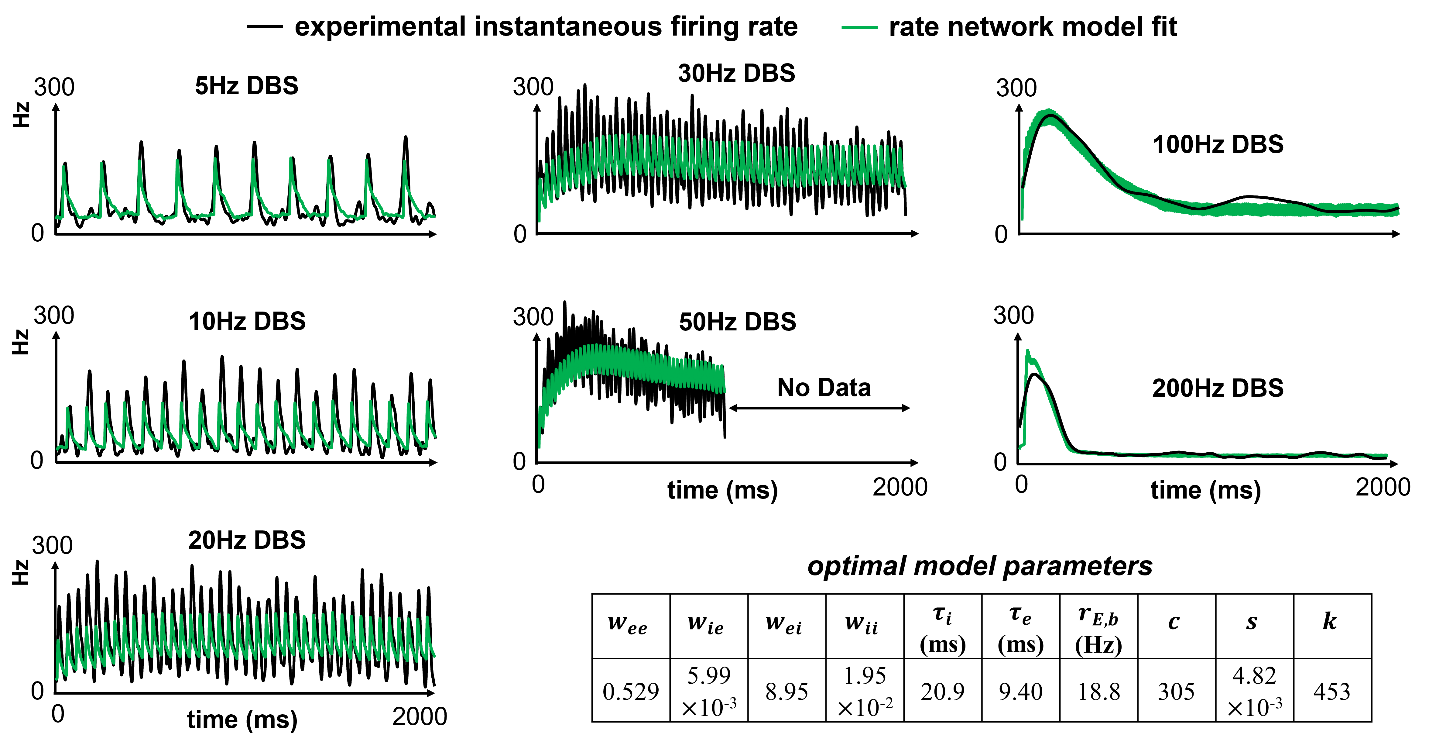


**Supplementary Figure 5. Fitting rate network model to Vim-DBS in humans**

*The firing rate network model is fitted to the experimental data recorded in the neurons in the ventral intermediate nucleus (Vim) of the human patients with essential tremor, during DBS of various stimulation frequencies (5 to 200Hz). For each frequency of DBS, we obtained 5 to 8 spike trains from experimental single-unit recordings in different patients, and compute the instantaneous firing rate using a time histogram method. In computing the experimental instantaneous firing rate, we implement an optimized Gaussian kernel that best characterized the Poisson process underlying the spiking data* [21]*. From the data of each DBS frequency, we present the model fit of the initial 2s; an exception is 50Hz DBS data, where the length of recording was ~1s. We compare the model fit (green line) with the experimental instantaneous firing rate (black line). The optimal model parameters (****Equation 15****) are obtained with our route optimization method* [4]*.* $w_{pq}$ *(*$p,q\in\left\{ i,e \right\}$*) is the connectivity strength (see the legend of* ***Supplementary Figure 4*** *for specific descriptions).* $\tau_{e}$ *and* $\tau_{i}$ *are the excitatory and inhibitory time constants, respectively.* $r_{E,b}$ *is the baseline firing rate of the external excitatory nuclei (group* $E$*,* ***Supplementary Figure 4****).* $c$, $s$ *and* $k$ *are the phenomenological parameters in the sigmoid transfer function* $F$ *(****Equation 15****).*

**
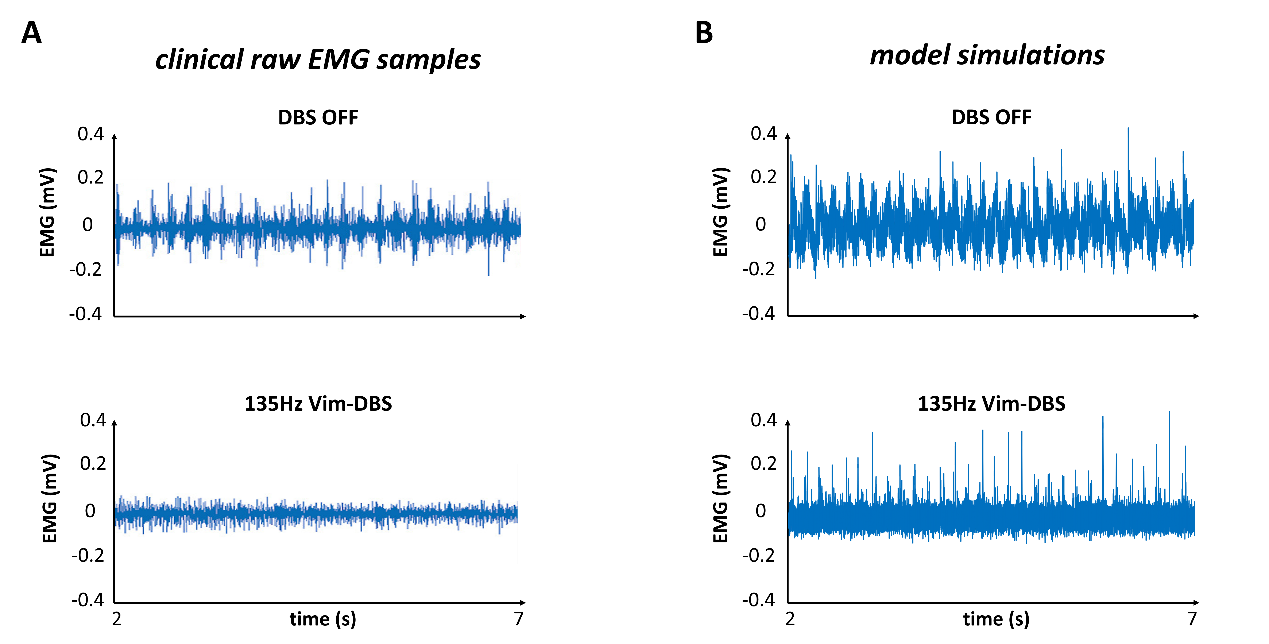
**

**Supplementary Figure 6. Compare clinical raw EMG samples and model simulations**

*The clinical raw EMG samples were recorded from the extensor of an essential tremor patient during DBS-OFF and 135-Hz Vim-DBS (data from Cernera et al. (2021)* [24]*). We compare these clinical data with the steady state EMG simulations from our model. The model simulations are presented in steady state (beyond 2 s), and are similar to these clinical data in terms of the EMG amplitudes. We observe a 4 – 6 Hz tremor in the clinical EMG sample with DBS-OFF; this is consistent with the corresponding model simulation. During 135-Hz Vim-DBS, in both clinical data and model simulation, we observe that the tremor activities are mostly suppressed. The tremor is suppressed to a higher extent during 135-Hz Vim-DBS in clinical data compared with the model simulation. However, there is high variability in EMG activities across different individuals, and thus the clinical Vim-DBS frequency need to be optimized specifically for individual ET patients* [25][24][26]*.*

**References**

[1] M. Tsodyks, K. Pawelzik, and H. Markram, “Neural networks with dynamic synapses,” *Neural Comput.*, vol. 10, no. 4, pp. 821–835, May 1998, doi: 10.1162/089976698300017502.

[2] L. Milosevic *et al.*, “A theoretical framework for the site-specific and frequency-dependent neuronal effects of deep brain stimulation,” *Brain Stimulat.*, vol. 14, no. 4, pp. 807–821, Jul. 2021, doi: 10.1016/j.brs.2021.04.022.

[3] Y. Tian *et al.*, “Modeling Instantaneous Firing Rate of Deep Brain Stimulation Target Neuronal Ensembles in the Basal Ganglia and Thalamus,” *Neuromodulation Technol. Neural Interface*, May 2023, doi: 10.1016/j.neurom.2023.03.012.

[4] Y. Tian *et al.*, “Uncovering network mechanism underlying thalamic Deep Brain Stimulation using a novel firing rate model.” bioRxiv, p. 2023.12.09.570924, Dec. 10, 2023. doi: 10.1101/2023.12.09.570924.

[5] Y. Wang, H. Markram, P. H. Goodman, T. K. Berger, J. Ma, and P. S. Goldman-Rakic, “Heterogeneity in the pyramidal network of the medial prefrontal cortex,” *Nat. Neurosci.*, vol. 9, no. 4, pp. 534–542, Apr. 2006, doi: 10.1038/nn1670.

[6] H. Markram *et al.*, “Reconstruction and Simulation of Neocortical Microcircuitry,” *Cell*, vol. 163, no. 2, pp. 456–492, Oct. 2015, doi: 10.1016/j.cell.2015.09.029.

[7] J. F. Marsden, P. Ashby, P. Limousin-Dowsey, J. C. Rothwell, and P. Brown, “Coherence between cerebellar thalamus, cortex and muscle in man: Cerebellar thalamus interactions,” *Brain*, vol. 123, no. 7, pp. 1459–1470, Jul. 2000, doi: 10.1093/brain/123.7.1459.

[8] M. Oswald, M. Tantirigama, I. Sonntag, S. Hughes, and R. Empson, “Diversity of layer 5 projection neurons in the mouse motor cortex,” *Front. Cell. Neurosci.*, vol. 7, 2013, Accessed: Mar. 02, 2022. [Online]. Available: https://www.frontiersin.org/article/10.3389/fncel.2013.00174

[9] S. Murray Sherman and R. W. Guillery, “Chapter II - The Nerve Cells of the Thalamus,” in *Exploring the Thalamus*, S. Murray Sherman and R. W. Guillery, Eds., San Diego: Academic Press, 2001, pp. 19–58. doi: 10.1016/B978-012305460-9/50016-2.

[10] N. E. Charalambakis, G. Govindaiah, P. W. Campbell, and W. Guido, “Developmental Remodeling of Thalamic Interneurons Requires Retinal Signaling,” *J. Neurosci.*, vol. 39, no. 20, pp. 3856–3866, May 2019, doi: 10.1523/JNEUROSCI.2224-18.2019.

[11] F. P. Chabrol, A. Blot, and T. D. Mrsic-Flogel, “Cerebellar Contribution to Preparatory Activity in Motor Neocortex,” *Neuron*, vol. 103, no. 3, pp. 506-519.e4, Aug. 2019, doi: 10.1016/j.neuron.2019.05.022.

[12] B. Benedetti, D. Dannehl, J. M. Janssen, C. Corcelli, S. Couillard-Després, and M. Engelhardt, “Structural and Functional Maturation of Rat Primary Motor Cortex Layer V Neurons,” *Int. J. Mol. Sci.*, vol. 21, no. 17, p. E6101, Aug. 2020, doi: 10.3390/ijms21176101.

[13] Y. Baumel, G. Jacobson, and D. Cohen, “Implications of functional anatomy on information processing in the deep cerebellar nuclei,” *Front. Cell. Neurosci.*, vol. 3, 2009, Accessed: Feb. 18, 2022. [Online]. Available: https://www.frontiersin.org/article/10.3389/neuro.03.014.2009

[14] J. E. Frandolig *et al.*, “The Synaptic Organization of Layer 6 Circuits Reveals Inhibition as a Major Output of a Neocortical Sublamina,” *Cell Rep.*, vol. 28, no. 12, pp. 3131-3143.e5, Sep. 2019, doi: 10.1016/j.celrep.2019.08.048.

[15] N. Yousif, P. G. Bain, D. Nandi, and R. Borisyuk, “A Population Model of Deep Brain Stimulation in Movement Disorders From Circuits to Cells,” *Front. Hum. Neurosci.*, vol. 14, 2020, doi: 10.3389/fnhum.2020.00055.

[16] S. Lim *et al.*, “Inferring learning rules from distributions of firing rates in cortical neurons,” *Nat. Neurosci.*, vol. 18, no. 12, Art. no. 12, Dec. 2015, doi: 10.1038/nn.4158.

[17] M. A. Dufour, A. Woodhouse, J. Amendola, and J.-M. Goaillard, “Non-linear developmental trajectory of electrical phenotype in rat substantia nigra pars compacta dopaminergic neurons,” *eLife*, vol. 3, Oct. 2014, doi: 10.7554/eLife.04059.

[18] B. K. Murphy and K. D. Miller, “Balanced amplification: a new mechanism of selective amplification of neural activity patterns,” *Neuron*, vol. 61, no. 4, pp. 635–648, Feb. 2009, doi: 10.1016/j.neuron.2009.02.005.

[19] O. Sporns, D. R. Chialvo, M. Kaiser, and C. C. Hilgetag, “Organization, development and function of complex brain networks,” *Trends Cogn. Sci.*, vol. 8, no. 9, pp. 418–425, Sep. 2004, doi: 10.1016/j.tics.2004.07.008.

[20] H. Shimazaki and S. Shinomoto, “A method for selecting the bin size of a time histogram,” *Neural Comput.*, vol. 19, no. 6, pp. 1503–1527, Jun. 2007, doi: 10.1162/neco.2007.19.6.1503.

[21] H. Shimazaki and S. Shinomoto, “Kernel bandwidth optimization in spike rate estimation,” *J. Comput. Neurosci.*, vol. 29, no. 1, pp. 171–182, 2010, doi: 10.1007/s10827-009-0180-4.

[22] G. M. Earhart, M. Hong, S. D. Tabbal, and J. S. Perlmutter, “Effects of Thalamic Stimulation Frequency on Intention and Postural Tremor,” *Exp. Neurol.*, vol. 208, no. 2, pp. 257–263, Dec. 2007, doi: 10.1016/j.expneurol.2007.08.014.

[23] S. C. Reitz *et al.*, “Comparing Programming Sessions of Vim-DBS,” *Front. Neurol.*, vol. 11, 2020, Accessed: Mar. 15, 2022. [Online]. Available: https://www.frontiersin.org/article/10.3389/fneur.2020.00987

[24] S. Cernera *et al.*, “Wearable sensor-driven responsive deep brain stimulation for essential tremor,” *Brain Stimul. Basic Transl. Clin. Res. Neuromodulation*, vol. 14, no. 6, pp. 1434–1443, Nov. 2021, doi: 10.1016/j.brs.2021.09.002.

[25] J. A. Herron, M. C. Thompson, T. Brown, H. J. Chizeck, J. G. Ojemann, and A. L. Ko, “Chronic electrocorticography for sensing movement intention and closed-loop deep brain stimulation with wearable sensors in an essential tremor patient,” *J. Neurosurg.*, vol. 127, no. 3, pp. 580–587, Sep. 2017, doi: 10.3171/2016.8.JNS16536.

[26] D. E. Vaillancourt, M. M. Sturman, L. Verhagen Metman, R. a. E. Bakay, and D. M. Corcos, “Deep brain stimulation of the VIM thalamic nucleus modifies several features of essential tremor,” *Neurology*, vol. 61, no. 7, pp. 919–925, Oct. 2003, doi: 10.1212/01.wnl.0000086371.78447.d2.
